# The genetic landscape of Scotland and the Isles

**DOI:** 10.1073/pnas.1904761116

**Published:** 2019-09-03

**Authors:** Edmund Gilbert, Seamus O’Reilly, Michael Merrigan, Darren McGettigan, Veronique Vitart, Peter K. Joshi, David W. Clark, Harry Campbell, Caroline Hayward, Susan M. Ring, Jean Golding, Stephanie Goodfellow, Pau Navarro, Shona M. Kerr, Carmen Amador, Archie Campbell, Chris S. Haley, David J. Porteous, Gianpiero L. Cavalleri, James F. Wilson

**Affiliations:** ^a^School of Pharmacy and Molecular and Cellular Therapeutics, Royal College of Surgeons in Ireland, Dublin D02 YN77, Ireland;; ^b^FutureNeuro Research Centre, Royal College of Surgeons in Ireland, Dublin D02 YN77, Ireland;; ^c^Genealogical Society of Ireland, Dún Laoghaire, Co. Dublin A96 AD76, Ireland;; ^d^Medical Research Council Human Genetics Unit, Institute of Genetics and Molecular Medicine, University of Edinburgh, Western General Hospital, Edinburgh EH4 2XU, Scotland;; ^e^Centre for Global Health Research, Usher Institute, University of Edinburgh, Edinburgh EH8 9AG, Scotland;; ^f^Bristol Bioresource Laboratories, Population Health Sciences, Bristol Medical School, University of Bristol, Bristol BS8 2BN, United Kingdom;; ^g^Medical Research Council Integrative Epidemiology Unit at the University of Bristol, Bristol BS8 2BN, United Kingdom;; ^h^Centre for Academic Child Health, Population Health Sciences, Bristol Medical School, University of Bristol, Bristol BS8 1NU, United Kingdom;; ^i^Private address, Isle of Man IM7 2EA, Isle of Man;; ^j^Centre for Genomic and Experimental Medicine, Institute of Genetics and Molecular Medicine, University of Edinburgh, Western General Hospital, Edinburgh EH4 2XU, Scotland;; ^k^The Roslin Institute and Royal (Dick) School of Veterinary Sciences, University of Edinburgh, Edinburgh EH25 9RG, Scotland

**Keywords:** population genetics, history, migration, fine-scale structure

## Abstract

Britain and Ireland are known to show population genetic structure; however, large swathes of Scotland, in particular, have yet to be described. Delineating the structure and ancestry of these populations will allow variant discovery efforts to focus efficiently on areas not represented in existing cohorts. Thus, we assembled genotype data for 2,554 individuals from across the entire archipelago with geographically restricted ancestry, and performed population structure analyses and comparisons to ancient DNA. Extensive geographic structuring is revealed, from broad scales such as a NE to SW divide in mainland Scotland, through to the finest scale observed to date: across 3 km in the Northern Isles. Many genetic boundaries are consistent with Dark Age kingdoms of Gaels, Picts, Britons, and Norse. Populations in the Hebrides, the Highlands, Argyll, Donegal, and the Isle of Man show characteristics of isolation. We document a pole of Norwegian ancestry in the north of the archipelago (reaching 23 to 28% in Shetland) which complements previously described poles of Germanic ancestry in the east, and “Celtic” to the west. This modern genetic structure suggests a northwestern British or Irish source population for the ancient Gaels that contributed to the founding of Iceland. As rarer variants, often with larger effect sizes, become the focus of complex trait genetics, more diverse rural cohorts may be required to optimize discoveries in British and Irish populations and their considerable global diaspora.

## Significance

Modern genetic analysis has revealed genetic differentiation across the south of Britain and Ireland. This structure demonstrates the impact of hegemonies and migrations from the histories of Britain and Ireland. How this structure compares to the north of Britain, Scotland, and its surrounding Isles is less clear. We present genomic analysis of 2,544 British and Irish, including previously unstudied Scottish, Shetlandic and Manx individuals. We demonstrate widespread structure across Scotland that echoes past kingdoms, and quantify the considerable structure that is found on its surrounding isles. Furthermore, we show the extent of Norse Viking ancestry across northern Britain and estimate a region of origin for ancient Gaelic Icelanders.

First documented in the fourth century BCE by the Greek explorer Pytheas, who describes its 3 corners (1), the archipelago of islands that includes Great Britain and Ireland has experienced an extensive history of migrations and invasions. After the initial Paleolithic settlement, there was migration of agriculturists around 4000–3000 BCE (2, 3), and then a population turnover associated with bronze and copper working and the Bell Beaker material culture (1, 3). With this establishment of the “Insular Atlantic” gene pool (2), subsequent migrations have influenced but not replaced the underlying haplotype diversity. The Anglo-Saxon invasions between 400 CE and 650 CE, for example, are associated with a higher German-related ancestry in the south of England (4, 5), and the Norse Viking incursions from the eighth to 11th centuries are associated with an increase of Norwegian-related ancestry into both Orkney (4, 6, 7) and Ireland (8, 9). In addition to these migrations, the northeast of Ireland also experienced admixture from Scottish and English sources that dates primarily to the Ulster Plantations of the 17th century (8, 9).

Previous genome-wide investigations of British (4) and Irish (8, 9) population genetics have undersampled Scotland and neighboring regions relative to England, Wales, and Ireland. Addressing this, we sought to combine samples from multiple cohorts in order to capture the majority of British and Irish diversity, including previously understudied regions, e.g., Scotland, the Hebrides, Shetland, and the Isle of Man. We combine data from both previously published sources and genotypes from Shetland, the Isle of Man, and the western coasts of Scotland, and analyze this comprehensive sample.

Using this sample, we sought to ask 3 questions: First, what is the fine-scale genetic structure of Scotland and its surrounding Isles? Second, using modern samples from Scandinavia, what is the Norse Viking contribution to these populations? Third, with the advent of ancient sampling of the Viking Period Gaelic settlers of Iceland, is it possible to trace their origins back to regions within Britain and Ireland?

## Results

### Genetic Landscape of Scotland and Ireland.

Addressing our first question of population structure, we assembled a combined dataset of 2,544 individuals, each with regional ancestry within the Isles. Individuals were sampled from a variety of cohorts and single nucleotide polymorphism array genotyping platforms, giving 341,924 common markers after quality control (see *Materials and Methods* and *SI Appendix*, *Supplementary Data 1*). Studies with comparable marker numbers have previously reported fine-scale structure in European-descent populations using haplotype information (9, 10). We utilized a number of analyses to study the genetic structure in Scotland, the rest of Britain, and Ireland, including haplotype-based fineSTRUCTURE clustering (11), dimension reducing t-distributed stochastic neighbor embedding (t-SNE) (12, 13) and principal component analysis, ADMIXTURE (14) analyses (see *Materials and Methods*), and Estimated Effective Migration Surface (EEMS) analysis. We used t-SNE, as it outperforms similar methods at reducing such data into lower-dimensional space, particularly when there is heterogeneous scaling, as is the case when both large-scale and local, fine-scale structure is present.

**Fig. 1.**
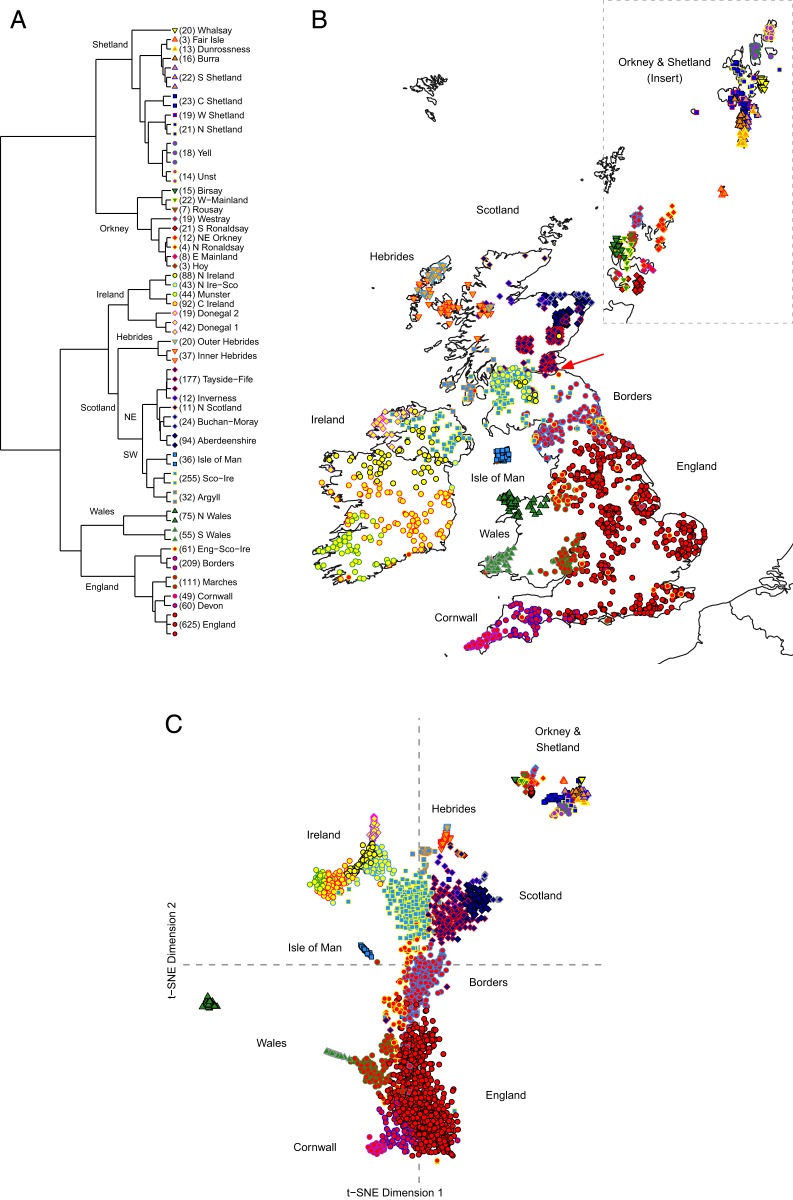
A comprehensive description of population structure across Britain and Ireland. (*A*) The dendrogram with each branch as one *k* = 65 final cluster identified by fineSTRUCTURE. Some clusters have been merged, resulting in *k* = 43 clusters. Cluster membership count is shown in parentheses. Merged clusters are indicated throughout by the same colored shape and merge label. (*B*) The average geographic position of 2,429 British and Irish individuals’ ancestors’ birthplaces, with cluster membership shown. The ancestry information available for some individuals’ ancestral place of birth are regional in resolution; therefore, a random jitter was introduced to these individuals’ positions. The exit of the Firth of Forth is indicated by the red arrow. The administrative boundaries of mainland Britain and Ireland were sourced from the R package, rworldxtra. (*C*) The genetic space positions of 2,544 British and Irish individuals as calculated in t-distributed stochastic neighbor embedding t-SNE analysis of the coancestry matrix obtained by ChromoPainter. Shown plotted are the first and second t-SNE dimensions. All plots were generated in R with the ggplots2 package.

Both fineSTRUCTURE and t-SNE reveal a remarkable degree of differentiation across the Isles. Our fineSTRUCTURE genetic clusters describe geographic regions within the Isles (Fig. 1 *A* and *B*), and t-SNE is essentially able to reconstruct the geographic locations of an individual’s ancestry based solely on their haplotype sharing (Fig. 1*C*). We label our fineSTRUCTURE clusters post hoc, in order to reflect the recent ancestral origins of their members. As some of the final 65 fineSTRUCTURE clusters (*SI Appendix*, *Supplementary Data 2*) were either too small or inadequately geographically labeled, we merged uninformative clusters into their nearest neighbor on the fineSTRUCTURE tree, leaving 43 “merged” fineSTRUCTURE clusters to further analyze. We denote cluster names in *italics* in order to differentiate them from the regions that they may be named after. Principal Component Analysis (PCA) and ADMIXTURE analyses demonstrate the primary sources of differentiation within the Isles (England/Wales versus Scotland/Ireland) and Orkney/Shetland versus the rest (*SI Appendix*, Fig. S2).

**Fig. 2.**
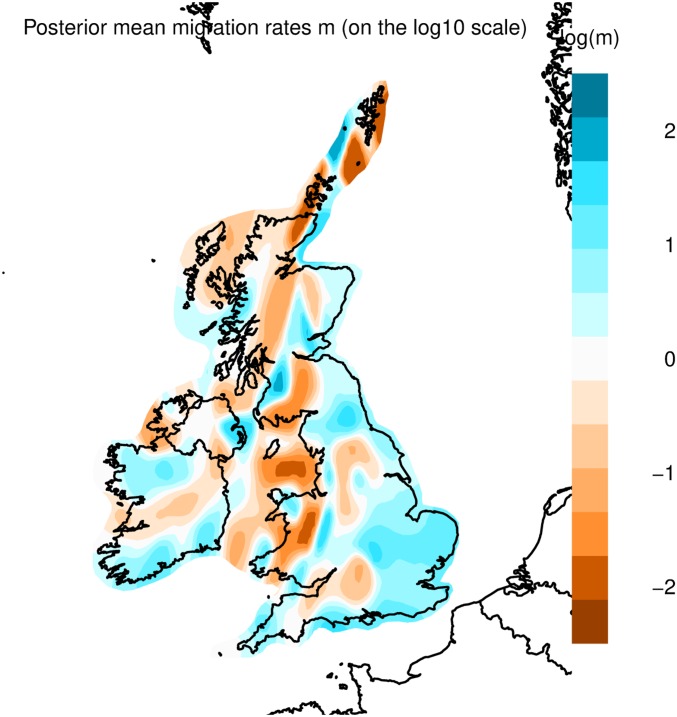
EEMS of Britain and Ireland. Shown are the posterior mean migration rates of 10 independent EEMS analytical runs (m, on a log10 scale). The image was produced using R and the rEEMSplots package, and the administrative boundaries and outline were sourced from the R package rworldxtra. Many strong barriers to migration can be seen in orange shades in Scotland, Wales, and Ireland.

We were able to categorize our expanded sample of Scotland into 6 groups of genetic clusters: the northeast, the southwest, the Borders, the Hebrides, Orkney, and Shetland (Fig. 1*A*). The majority of Scottish samples are placed in either the northeast or the southwest group, reflecting a previously observed cline within Scotland (15), and the primary mainland structure within Scotland (4). This split appears to be geographically centered near the River Forth, which empties into the Firth of Forth (Fig. 1*B*). For more detail of the geography of the River Forth, see *SI Appendix*, Fig. S3. This genetic division is apparent in our fineSTRUCTURE dendrogram (Fig. 1*A*), the first t-SNE dimension (Fig. 1*C*), and in our migration surface analysis (Fig. 2). ADMIXTURE analysis (*SI Appendix*, Fig. S2*B*) shows a similar profile across this cline, suggesting the primary source of differentiation is isolation by distance.

The northeast of Scotland is dominated by 2 large clusters whose ancestry originates from the main administrative regions of the area, *Tayside-Fife* and *Aberdeenshire*. It also includes the small cluster *Buchan-Moray* which exhibits characteristics of isolation: high fixation indices (F_ST_) (Dataset S1) and high levels of short and long runs of homozygosity (ROH) (*SI Appendix*, Fig. S4 and *Supplementary Data 3*), with a gene flow barrier in EEMS (Fig. 2). The southwest Scottish branch includes samples with ancestry from the western coast area of Argyll and the Isle of Man, but the majority belong to the *Sco-Ire* cluster. Members of the *Sco-Ire* cluster have ancestries either from the northeast of Ireland or the southwest of Scotland. The distribution of Irish members of *Sco-Ire* appears to be limited to regions of north Ireland which saw substantial plantation of British migrants in the 17th century.

The southern, Borders, region of Scotland forms the boundary between Scotland and England. Individuals with ancestry from this region separate from other Scottish clusters and instead cluster with individuals of English ancestry (*Borders*). This border phenomenon is reflected in our EEMS analysis (Fig. 2 and *SI Appendix*, *Supplementary Data 4*), where we observe a genetic barrier separating the Borders from the rest of Scotland.

The north of Scotland is a sparsely populated, mountainous region known as the Highlands. This isolation is demonstrated in our migration analysis (Fig. 2) which shows a gene flow barrier separating it from the remainder of Scotland. We observe 2 distinct, small fineSTRUCTURE clusters of individuals with Highland ancestry, *N Scotland* and *Inverness*, placed on the *Aberdeenshire and Tayside-Fife* fineSTRUCTURE branches, respectively. Recent gene flow from Tayside-Fife to the less isolated *Inverness*, or low sampling coverage, could impact cluster branching and explain this separation. In PCA analysis, *N Scotland* forms a genetic continuum located in between Scotland and the Northern Isles (*SI Appendix*, Fig. S2*A*), and presents higher Northern Isles ancestry components in ADMIXTURE analysis (*SI Appendix*, Fig. S2*B*). This suggests part of *N Scotland*’s differentiation is due to shared ancestry with the Northern Isles.

The Hebrides also appear genetically distinct from the rest of mainland Scotland. Hebridean individuals are separate from the other Scottish populations studied in PCA (*SI Appendix*, Fig. S2*A*). In t-SNE analysis (Fig. 1*C*), the Hebrides forms a discrete “island” within the t-SNE dimensions. This isolation suggests a small, isolated, ancestral island population differentiated by isolation by distance. This is further supported by an increase in long (>5 Mb) ROH (16) (*SI Appendix*, Fig. S4) and the low gene flow rates estimated via EEMS (Fig. 2). Hebridean individuals present genetic affinities to both the north of Scotland (where they are geographically located) and to Ireland; firstly, Hebrideans separate from other western Scottish samples along principal component 1 and instead cluster with *N Scotland* individuals (*SI Appendix*, Fig. S3*A*), and, secondly, Hebridean samples demonstrate Irish affinity along principal component 2 and have a higher proportion of ADMIXTURE ancestral components most frequent in Ireland (*SI Appendix*, Fig. S2). Interestingly, the cluster *Argyll* appears intermediate between *Sco-Ire* (which it shares a fineSTRUCTURE branch with) and the Hebrides; *Argyll* plots in between the Hebrides and *Sco-Ire* in t-SNE analysis (Fig. 1*C*), and *Argyll* individuals present similar principal component 2 coordinates and ADMIXTURE ancestry proportions to the Hebrides (*SI Appendix*, Fig. S2).

Orkney and Shetland are the most differentiated populations in our sample of the British Isles and Ireland, in agreement with previous observations (4, 6, 17–19). Orkney and Shetland show high F_ST_ (Dataset S1), extremely low gene flow (Fig. 2), and the highest burden of ROH in our sample (*SI Appendix*, Fig. S4). They form their own isolated “islands” in t-SNE analysis, and fineSTRUCTURE clustering reveals that the Northern Isles are the first branch in the tree separating from all other British and Irish populations. This suggests that, in addition to Norwegian-related admixture (4), differentiation in the Northern Isles has also been driven by isolation. Our clustering analysis captures a remarkable degree of differentiation within each island group, separating individual Isles (*SI Appendix*, *Supplementary Discussion*).

Beyond Scotland, we describe the genome-wide genetics of the Isle of Man, as well as capturing novel structure in the northwestern Irish county of Donegal. The Isle of Man is grouped with the southwestern Scottish individuals, and Donegal appears to be at the end of a genetic axis within Ireland. We discuss these results in greater detail in *SI Appendix*, *Supplementary Discussion*.

### Norwegian Ancestry in Britain and Ireland.

Our sample of Britain and Ireland covers the historical range of Norse Viking influence in the north of Britain, and Ireland. Thus, to answer our second question, we investigated the extent of Norwegian ancestry in fineSTRUCTURE clusters whose geographic ranges overlapped with the historical range of Norse Viking activity. We investigated using 2 different methods, utilizing our dataset of 2,554 British and Irish individuals together with 2,225 additional Scandinavians. For more information about these Scandinavians, see *SI Appendix*, *Supplementary Data 6*.

**Fig. 3.**
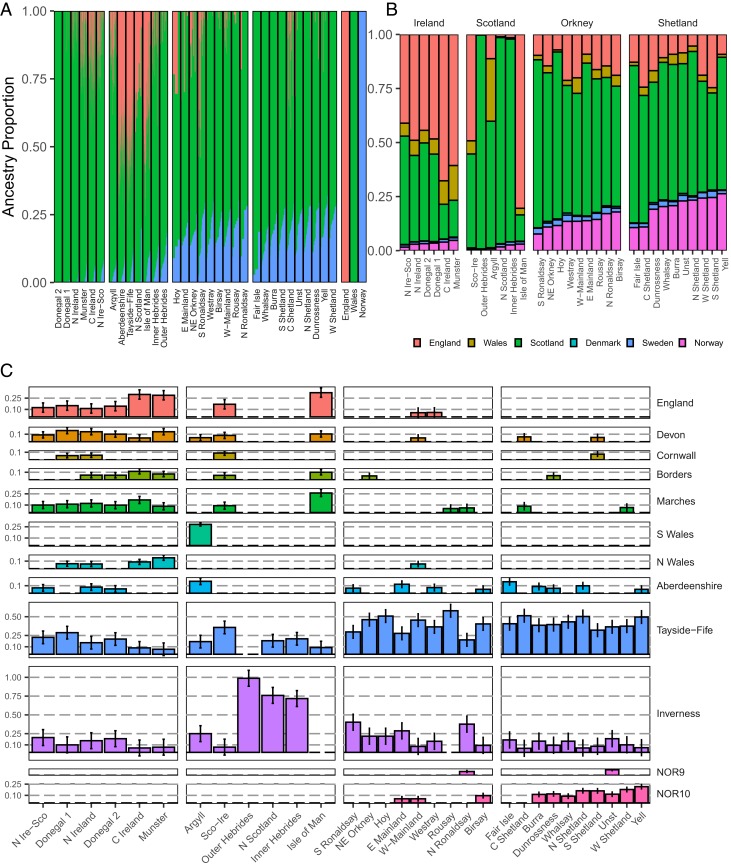
Norwegian Ancestry across Britain and Ireland. (*A*) Ancestry proportions of a supervised ADMIXTURE analysis, using 625 English, 130 Welsh, and 893 Norwegians as references and select fineSTRUCTURE clusters as test individuals. (*B*) The mean total genome-wide proportion of haplotype contributions of reference British and Scandinavian populations to tested British and Irish fineSTRUCTURE clusters. (*C*) The mean genome-wide proportions of haplotype contributions of reference British and Scandinavian reference clusters (*y* axis) that contribute ≥5% of the total proportion to any one individual target cluster (*x* axis). Error bars indicate the 95% confidence intervals calculated from the 200 sampled iterations of the SOURCEFIND analysis.

We first performed a supervised ADMIXTURE (14) analysis, modeling British and Irish clusters of interest as a mixture of 3 sources, *England*, ‘Wales’ (*N Wales* and *S Wales*), and Norway (Fig. 3*A*). These sources approximately represent Celtic (Wales), Saxon (England), and Norse (Norway). We observe the highest Norwegian ancestry in the Northern Isles clusters (mean 18%, maximum 23%), which agrees well with estimates by Leslie et al. (4). Norwegian ancestry is lower in the Hebrides (7%), and is substantially lower in the north of Scotland (*N Scotland*) and southwest (*Argyll*), and the Isle of Man, averaging 4%, little more than other parts of mainland Scotland. We estimate that Norwegian (as well as Danish/Swedish) ancestry is also markedly low in Ireland (average 7%) compared with previous estimates (8, 9) (we explore this further in *Discussion*). The majority of ancestry in our tested clusters is modeled as the Welsh ancestral component, reflecting a common “Celtic” ancestry across Scotland and Ireland. The eastern Scottish clusters *Aberdeenshire* and *Tayside-Fife* present more English-like ancestry. *Isle of Man* similarly presents relatively high (42%) English ancestry.

We compared the ADMIXTURE results to those of SOURCEFIND (20), a haplotype-based analysis with greater sensitivity. We modeled the genomes of the British and Irish fineSTRUCTURE “target” clusters as a mixture of haplotypes from other British, Norwegian, Danish, or Swedish (i.e., “Scandinavian”) reference clusters (*SI Appendix*, *Supplementary Data 6*). We calculated the mean total ancestry contributions from reference to target (Fig. 3*B*) and the proportions of those reference clusters whose haplotypes account for more than 5% of the ancestry of any one target cluster (Fig. 3*C*). Mirroring the ADMIXTURE analysis, we observe the highest levels of Norwegian-like haplotypes in Shetland, then Orkney, followed by the Hebrides, the Isle of Man, and Ireland (Fig. 3*B*). Our estimates of Norwegian ancestry across northern Britain are in agreement with our ADMIXTURE analysis. We estimate the total of Norwegian-like ancestry in Orkney and Shetland to be about 20 to 25%. The proportion of Norwegian-like ancestry also tends to be higher in individuals with ancestry from the north of Orkney and of Shetland. The largest source of Norwegian-like haplotypes in Britain and Ireland comes from 2 clusters, NOR9 and NOR10, predominantly consisting of individuals sampled from the western Norwegian counties of Hordaland and Sogn & Fjordane (Fig. 3*C*), the area whence most Norse Vikings set sail.

### Ancient Genetic Links.

We reasoned that the isolated regions in the north of Britain and Ireland may act as proxies for the historical populations of these regions—which are as yet unsampled with ancient DNA. Therefore, we set out to identify possible British or Irish source regions for previously reported ancient Gaels from Iceland (21). We estimated shared genetic drift between these 27 ancient Icelanders and modern British and Irish populations we have described and modern Scandinavians we have used elsewhere in this study.

**Fig. 4.**
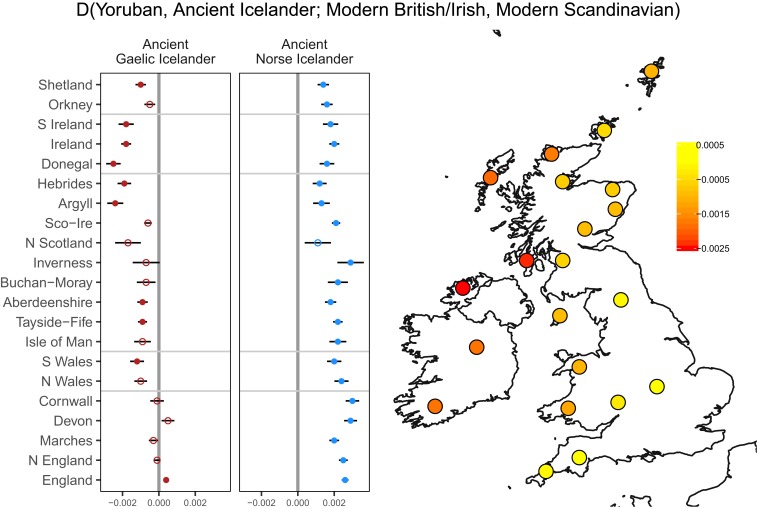
Shared drift between ancient Gaels and modern British and Irish populations. The *D* statistics of ancient predominantly Gaelic (*n* = 7) and predominantly Norse (*n* = 10) Icelanders, comparing affinity to either modern British or Irish genetic regions and modern Scandinavia. A negative *D* statistic indicates more shared drift with Britain or Ireland, and a positive value indicates more shared drift with modern Scandinavia. Shown are the estimates with SEs (*Left*), with estimates with a |Z| > 3 shown as filled circles and |Z| < 3 shown as hollow circles. We also show the values of the ancient Gaelic Icelander *D*-statistic estimates for each modern British or Irish region mapped to the general geographic position of that cluster(s). This was plotted in the statistical computing language R (39) and the packages ggplots2 and rworldxtra.

We first confirmed that our underlying modern British and Irish structure does not dramatically change the ancestry estimates of the ancient Icelanders (*SI Appendix*, *Supplementary Data 7*). Using Human Genome Diversity Project (HGDP) Yorubans as an outgroup, we calculated the *D* statistic of 2 groups of ancient individuals to either British or Irish genetic regions, or modern Scandinavia (Fig. 4). The British or Irish regions included some merged fineSTRUCTURE clusters in Orkney, Shetland, and the Hebrides, Donegal (*Donegal 1* and *2*), Ireland (*N Ireland*, *C Ireland*), and S Ireland (*Munster*) to increase sample sizes of those regions. The 2 groups of ancient Icelanders were those of predominantly Gaelic (*n* = 7) or Norse (*n* = 10) ancestry (see *SI Appendix*, *Supplementary Data 7* for more information). All but one of the predominantly Norse ancient Icelanders share significantly (Z > 3) more drift with modern Scandinavian individuals than with the British sample (Fig. 4). The predominantly Gaelic ancient Icelanders show differing affinities across the British and Irish groups. They show the greatest affinity to the “Gaelic” populations of Scotland and Ireland as opposed to English regions (Fig. 4). The smallest estimates of *D*, which correspond to the largest British/Irish affinity compared to Scandinavia, are to Donegal, the Hebrides, and Argyll. This corresponds to the northwestern region of the British Isles and Ireland which is known to have experienced heavy Viking activity (1). Interestingly we observe some ancient Gaelic Icelanders, in contrast, share affinity with different clusters (*SI Appendix*, Fig. S8), notably KNS-A1, who shows greater affinity to the south of Ireland. Our results provide genetic evidence either of Viking-mediated migration of Gaels from the northwest of the British Isles and Ireland or, at least, that these modern regions represent the best proxy of the true ancient Gaelic source populations in the absence of direct ancient DNA sampling.

## Discussion

Pytheas of Massalia’s famous work, *On the Ocean*, *ca*. 325 BCE, describes the 3 corners of Britain: *Kantion* (Kent), *Belerion* (Cornwall), and *Orkas* (Orkney). Previous sampling of the British Isles followed this lead with the 1000 Genomes Project British in England and Scotland (GBR) sampling these exact areas. With extensive representation across the archipelago, however, we describe the full extent of the northerly Norse pole of ancestry, adding to the known Saxon and Celtic poles in the southeast and west (4), respectively. The broad structure that we observe in Scotland and Orkney was also detected by the People of the British Isles study (4); however, with increased coverage across lowland Scotland, the Hebrides, and the Highlands, we reveal novel fine-scale genetic structure, and describe the genetics of Shetland and the Isle of Man. In addition, we reveal genetic structure reflecting the geography of the Isles at orders of magnitude finer scales than the mirroring of geography seen thus far in continental Europe (22).

The modern genetic landscape of the Isles reflects splits in the early languages of the Isles: Q-Celtic (Scottish, Irish and Manx Gaelic) and P-Celtic (Welsh, Cumbric, Cornish, Old Brythonic, Pictish). Scotland, the main focus of our analysis, is defined by a southwest versus northeast division near the River Forth (geographically located between the *Tayside-Fife* and *Sco-Ire* clusters in Fig. 1*A*). This division also echoes the historical distributions of Gaels versus Picts (see *SI Appendix*, Fig. S9 for a distribution of Pictish place names). The entire northeast branch of fineSTRUCTURE clusters describes the boundaries of the Pictish kingdoms, with the southwest branch mapping the Dark Age kingdoms of Strathclyde (*Sco-Ire*) and Dál Riata (*Argyll*). The *Borders* cluster coincides geographically with the Brythonic kingdoms of the Gododdin (modern Lothian and Borders) and Rheged (modern Cumbria). The legacy of the later Norse Jarldom of Orkney and its Scandinavian admixture drives the differentiation of the Northern Isles [for distributions of relevant historical groups, see Leslie et al. (4)]. Studies of ancient genomes are required to shed further light on the links between this modern structure and these groups.

We have further explored the impact of the Norse Viking migrations on Britain and Ireland and the geographic sources of ancient Gaelic settlers of Iceland. Our estimates of Norwegian ancestry in Ireland contrast starkly with previous estimates (4, 8, 9) which were much higher. While methodologies overlap and are related, we include British references in our analyses. With British and Scandinavian references, we find agreement across both ADMIXTURE and the haplotype-based methods, which employ subtly different marker information—either allele frequencies or haplotypes. Our estimates are also in better accord with Irish Y-chromosome data, which show little trace of Norse patrilineal ancestry in the modern Irish (23). Future use of rare variation from whole genome sequencing may provide a more direct and discerning method to quantify the extent of Norse admixture into Ireland. We also investigated this historical period utilizing ancient genomes of Gaelic settlers in Iceland who date to its founding. Although greater sampling with high-coverage genomic data is required to elucidate the individual origins of these settlers, and despite subsequent migration to and from Ireland and southwest Scotland since the Norse Viking period, our results cautiously suggest that the northwest peripheries of Britain and Ireland are the best modern proxies for their homeland.

Our results also may have implications for rare disease variant discovery within Britain and Ireland, where disease incidence is known to vary geographically (24). The extraordinary fine-scale haplotype diversity revealed here across the archipelago, particularly in Scotland and the surrounding Isles, is unlikely to be well represented in urban studies such as UK Biobank. Equitable translation of genomic findings into medicine may require that the full gamut of populations is well represented in genomic studies, both on a global scale (25) and also more locally within countries which show significant structure. Otherwise, these important low-frequency variants, which show larger effect sizes (26), will remain undiscovered or poorly characterized. Studies correlating the distribution of rare genetic variants with the structure defined by common variation will clarify the extent of the issue. Isolated and differentiated populations such as the ones we describe in the Hebrides, Argyll, the Scottish Highlands, or Donegal in Ireland, in particular, have many characteristics of utility for understanding the genetic basis of complex traits (27–29).

## Materials and Methods

For a full description of all of the methods and materials, see *SI Appendix*, *Supplementary Data 1*. We describe the methods and materials in brief here. We assembled a combined dataset of 2,554 individuals from 5 different cohorts of regional English, Welsh, Scottish, Manx, or Irish ancestry. We cleaned this combined dataset, controlling for missingness, minor allele frequency, relatedness, and markers from the human leukocyte antigen region. We performed fineSTRUCTURE (11) analysis on this combined dataset, as well as ADMIXTURE (14) and F_ST_ analysis on the dataset after markers had been pruned for excess linkage disequilibrium. We explored Norwegian ancestry in northern Britain using supervised ADMIXTURE (14) analysis and SOURCEFIND (20) analysis with 2,225 additional Scandinavian individuals. Lastly, using ancient Icelanders, we explored genetic affinities between modern British or Irish regions and ancient Icelanders using *D* statistics (30).

All participants in all studies gave written informed consent. Ethical approval for the GS:SFHS study was obtained from the Tayside Committee on Medical Research Ethics (on behalf of the National Health Service) ref: 05/S1404/89. GS:SFHS is a Research Tissue Bank, approved by the East of Scotland Research Ethics Service ref: 15/ES/0040. Ethical approval for SCOTVAR was from the Multi-Centre Research Ethics Committee for Scotland: MREC/00/0/17: Investigation of genetic characteristics of Scottish regional populations to assess their genetic ancestry and their suitability for genetic association studies. Favourable opinions are held VIKING from the South East Scotland Research Ethics Committee (12/SS/0151), for the Orkney Complex Disease Study (ORCADES) from the North of Scotland Research Ethics Committee (12 December 2003), for the Irish DNA Atlas from the Royal College of Surgeon Research Ethics Committee (REC0020563). Ethical approval for the study of the samples from the Isle of Man was given by the Isle of Man Ethical Committee on 17 January 1997.

## Supplementary Material

Supplementary File

Supplementary File

Supplementary File

Supplementary File

Supplementary File
