# Supplementary material for: The genetic landscape of Scotland and the Isles
Source: Proc Natl Acad Sci U S A. 2019 Sep 3;116(38):19064–70. doi: 10.1073/pnas.1904761116 (PMC6754546; doi:10.1073/pnas.1904761116)
Supplement: Supplementary File [file pnas.1904761116.sapp.pdf]

Supplementary Information for

The Genetic Landscape of Scotland and the Isles

Edmund Gilbert[1,2], Seamus O'Reilly[3], Michael Merrigan[3], Darren McGettigan[3], Veronique Vitart[4], Peter K Joshi[5], David W Clark[5], Harry Campbell[5], Caroline Hayward[4], Susan M Ring[6,7], Jean Golding[8], Stephanie Goodfellow[9], Pau Navarro[4], Shona M Kerr[4], Carmen Amador[4], Archie Campbell[10], Chris S Haley[4,11], David J Porteous[10], Gianpiero L Cavalleri[1,2]*, James F Wilson[4,5]*‡.

1. School of Pharmacy and Molecular and Cellular Therapeutics, Royal College of Surgeons in Ireland, 123 St Stephen's Green, Dublin, Ireland. **2.** FutureNeuro Research Centre, Royal College of Surgeons in Ireland, Dublin, Ireland. **3.** Genealogical Society of Ireland, Dún Laoghaire, Ireland. **4.** MRC Human Genetics Unit, Institute of Genetics and Molecular Medicine, University of Edinburgh, Western General Hospital, Edinburgh, EH4 2XU, Scotland. **5.** Centre for Global Health Research, Usher Institute, University of Edinburgh, Edinburgh, EH8 9AG, Scotland. **6.** Bristol Bioresource Laboratories, Population Health Sciences, Bristol Medical School, University of Bristol, BS8 2BN, Bristol, UK. **7.** Medical Research Council Integrative Epidemiology Unit at the University of Bristol, BS8 2BN, Bristol, UK. **8.** Centre for Academic Child Health, Population Health Sciences, Bristol Medical School, University of Bristol, BS8 1NU, Bristol, UK. **9.** *Private Address*, Isle of Man. **10.** Centre for Genomic and Experimental Medicine, Institute of Genetics and Molecular Medicine, University of Edinburgh, Western General Hospital, Edinburgh, EH4 2XU, Scotland. **11.** The Roslin Institute and Royal (Dick) School of Veterinary Sciences, University of Edinburgh, Edinburgh, EH25 9RG, Scotland.

* These authors contributed equally.
‡ Corresponding Author

Email: jim.wilson@ed.ac.uk

This PDF file includes:

- Supplementary text
- Figures S1 to S9
- Legends for Datasets S1 to S4
- SI References

Other supplementary materials for this manuscript include the following:

- Datasets S1 to S4

Genetic Ancestry of Scotland and the Isles

SI Appendix

Table of Contents

Supplementary Data 1 – Methods and Materials 3
Samples 3
Quality Control 4
Genetic Landscape of Scotland and Ireland 4
Norwegian Ancestry in Britain and Ireland 5
Ancient Genetic Links 5
Supplementary Data 2 – Final fineStructure Dendrogram 7
Supplementary Data 3 – Runs of Homozygosity 11
Methods and Results 11
Supplementary Data 4 – Estimate Effective Migration Surface Analysis 14
Methods and Results 14
Supplementary Data 5 – Isle of Man Population Structure 16
Methods and Results 16
Supplementary Data 6 – European Reference Clusters 18
Supplementary Data 7 – Ancient Genetic Links 19
Supplementary Discussion – Genetic Structure and Ancestry Across Britain and Ireland 23
Acknowledgments 26
Supplementary Table Legends 27
Dataset S1 27
Dataset S2 27
Dataset S3 27
Dataset S4 27
SI References 28

Supplementary Data 1 – Methods and Materials

Samples

We combined a number of datasets to construct a comprehensive sample of modern British and Irish genetic ancestry. Our study focused on a combined dataset of 2,554 modern samples consisting of individuals with extended ancestry originating from a specific region within the archipelago of Britain and Ireland. Briefly, this combined sample included; 1078 English, 131 Welsh, 40 Manx, 398 Irish, 57 Hebridean, 111 Orcadian, 172 Shetlandic, and 567 other Scottish, individuals. These individuals were sampled from the following cohorts; Generation Scotland[1,2] (n=551), Scotvar[3] (n=34), People of the British Isles[4] (n=1,308) European Longitudinal Study of Pregnancy and Childhood in the Isle of Man (ELSPAC IOM) study[5] (n=40), Irish DNA Atlas[6] (n=194), ORCADES[7] (n=111), and Viking Health Study – Shetland (VIKING) (n=172).

The majority of our Scottish samples (491/624) are from the Generation Scotland: Scottish Family Health Study cohort (GS) , and have previously been reported[1,2]. These samples were chosen on the basis that all four of their grandparents were born in the same Council Area of Scotland. We excluded urban individuals who met this criterion, but whose ancestors were born within the city administration regions of Aberdeen, Dundee, Edinburgh, or Glasgow. A smaller subset of individuals with Scottish ancestry (n=34) were sampled from the SCOTVAR collection[3] and were genotyped as part of this study. These individuals similarly were recruited on the basis that their four grandparents were born from the same region, either the Isle of Lewis, the Isle of Skye, or the Kintyre peninsula on the west of Scotland. Finally the remaining Scottish individuals (n=99) were sampled from the WTCCC People of the British Isles (PoBI) study[4], whose sampling criteria was that all four of their grandparents were born within 80 km. We obtained individual latitude and longitude data for all PoBI samples with a Data Access Agreement with the People of the British Isles Study. The samples from Wales (n=131) and England (n=1078) were solely sampled from the PoBI study and had the same sampling criteria as the PoBI Scottish samples. The Isle of Man samples (n=40) were obtained from the European Longitudinal Study of Pregnancy and Childhood in the Isle of Man (ELSPAC IOM) study[5], and were sampled on the basis that each individuals' four grandparents were born on the island. These genotypes were generated as part of this study. The plurality of the Irish samples (n=194/398) were sampled from the Irish DNA Atlas cohort, and have previous been reported.[6] The Irish DNA Atlas individuals were recruited on the basis that all eight great-grandparents were born within a 50 km region. Additional Irish samples (160) were obtained from the GS cohort, sampling individuals with all four grandparents from Ireland. A subset of these GS Irish were recontacted for additional genealogical information, allowing the geographic mapping of their ancestry. The remaining Irish individuals (44) were sampled from the PoBI cohort, and followed the same recruitment scheme as the other PoBI samples. All the Orcadian individuals (111) within this study were samples from the ORCADES study[7], each with all four grandparents born with the same parish in Orkney. All Shetland individuals included (172) were sampled from the Viking Health Study – Shetland (VIKING), with the same grandparental criteria as in Orkney, and the genotypes have not previously been reported.

All participants in all studies gave written informed consent. Ethical approval for the GS:SFHS study was obtained from the Tayside Committee on Medical Research Ethics (on behalf of the National Health Service) ref 05/S1404/89. GS:SFHS is a Research Tissue Bank, approved by the East of Scotland Research Ethics Service ref:15/ES/0040. Ethical approval for SCOTVAR was from the Multi-Centre Research Ethics Committee for Scotland: MREC/00/0/17: Investigation of genetic characteristics of Scottish regional populations to assess their genetic ancestry and their suitability for genetic association studies. Favourable opinions are held VIKING from the South East Scotland Research Ethics Committee (12/SS/0151), for ORCADES from the North of Scotland Research Ethics Committee (12/12/2003), for the Irish DNA Atlas from the Royal College of Surgeon Research Ethics Committee (REC0020563). Ethical approval for the study of the samples from the Isle of Man was given by the Isle of Man Ethical Committee on the 17th January 1997.

All GS, VIKING, SCOTVAR and Isle of Man individuals were genotyped on the Illumina OmniExpress array at the Edinburgh Clinical Research Facility, as were all ORCADES samples used here, according to manufacturer's instructions. DNA from the Isle of Man samples was extracted from whole blood collected in EDTA tubes using a salting out method[8], DNA was quantified using picogreen as described previously[9]. The PoBI individuals were genotyped on a custom Illumina 1.2M SNP array. The Irish DNA Atlas samples were genotyped on a mixture of the Illumina OmniExpress, and OmniExpress Exome SNP arrays.

The European samples were obtained from a WTCCC Multiple Sclerosis case-control dataset[10]. We obtained a subset of individuals who were recruited from 3 Scandinavian countries (Norway, Sweden, and Denmark). We extracted a subset of European ancestry using the same method described previously[6] – briefly we performed principal component analyses on the whole subset, and each country individually, with smartpca[11,12], removing outliers as per the default parameters. This left 2,225 individuals with Scandinavian genetic ancestry. Further geographic data were available on a subset of these European individuals (1,739/2,225), allowing the mapping of them to administrative regions within the countries of recruitment. Ancient Gael samples were obtained from previously published data[13], and were provided by the authors in plink[14,15] format. Genotype data for 21 Yoruban individuals from the Human Genome Diversity Project[16] were downloaded from publicly accessible datasets in plink[14,15] format.

Quality Control

Assembling a combined dataset of individuals with British and Irish ancestry, we took the intersection of markers between the GS, Irish DNA Atlas, PoBI, ORCADES, VIKING, SCOTVAR, and ELSPAC cohorts. Using plink[14,15] we merged the datasets, using only the markers common between all datasets. We excluded markers with >5% missingness (n=384), <2% minor allele frequency (n=416). As our analyses included Multiple Sclerosis cases, we excluded markers found at the HLA region on chromosome 6, a region with associations to that disease[10]. Lastly we excluded A/T or C/G SNPs. We additionally excluded individuals with >5% missingness, and closely related individuals – defining such as individuals with a plink pihat score > 0.09. We ignored this last, relatedness, criteria for individuals from Fair Isle as the remaining, native, population is under 10 individuals, and the basic level of relatedness is elevated.

For analyses combining continental European data, we took an intersection of the 341,924 markers in our British and Irish combined dataset and the 580,030 markers in the European dataset. The European dataset was subject to the same marker, and sample, quality control filters as the combined British and Irish dataset. This left a final intersection of 221,958 common markers between our British and Irish dataset, and the European dataset.

Genetic Landscape of Scotland and Ireland

To investigate genetic structure within the north of Britain and Ireland we combined samples from the GS, Irish DNA Atlas, PoBI, ORCADES, VIKING, SCOTVAR, and ELSPAC cohorts to give a combined dataset of 1078 English, 131 Welsh, 40 Manx, 398 Irish, 624 Scottish, 111 Orcadian, and 172 Shetlandic samples. This British and Irish combined sample consisted of 341,924 common markers and 2,554 individuals.

Using the combined dataset of 2,554 British and Irish individuals we performed fineStructure[17] analysis. We first phased the samples using SHAPEIT v2r790[18] and human genome build 37 – with default parameters with the exception of N_e which we set as 11,418 as recommended by the authors for European populations. We converted this SHAPEIT-formatted phased data to the ChromoPainter format using scripts provided by the authors of ChromoPainter/fineStructure. We then performed ChromoPainterv2 analysis, generating a haplotype "counts" co-ancestry matrix modelling every individual as a mixture of haplotypes donated from every other individual in the analysis. We used the default parameters, with the exception that we assumed 50 haplotype "chunks" per region, due to the longer haplotypes in Britain and Ireland[19]. With this co-ancestry matrix we performed fineStructure Markov Chain Monte Carlo (MCMC) clustering analysis, using 2M burnin iterations and 2M sampling iterations – sampling 500 of those iterations. Using the MCMC sample with the highest posterior probability we performed 100,000 additional hill climbing and dendrogram building moves to reach the final inferred clustering and dendrogram of clusters. When tree building we utilised the -T 1 parameter which uses the Maximum Concordance State method first reported by Leslie et al[19].

fineStructure identified 65 fine-scale clusters. These clusters were further represented in a dendrogram tree by fineStructure. These clusters ranged from 1 individual (two separate clusters of English outliers) to 623 individuals (a cluster containing the majority of individuals with English ancestry). Some of the clusters identified were either; too small to be informative, had already been described in detail by previous authors[6,19,20], or we did not have complete or precise grandparental place of birth information for individuals within that cluster. We therefore merged these ambiguous

clusters with the closest cluster on the same branch to summarise that branch together as one "merged" cluster. We focussed on the resolution of structure represented by the resulting 43 merged clusters.

We projected the ChromoPainter co-ancestry matrix into lower dimensional space using t-distributed stochastic neighbour embedding (t-SNE)[21,22]. We used R[23] (version 3.5.0) package Rtsne to perform the t-SNE analysis on the co-ancestry matrix with 5,000 iterations using a perplexity of 30, a learning rate of 200 and an initial PCA calculated over 100 dimensions. We found that these parameters were able to visualise the genetic data without spurious artefacts from the t-SNE algorithm. We plotted the dimensions using the ggplot2 R package.

In addition to the t-SNE analysis, we performed principal component analysis on the ChromoPainter haplotype sharing "chunkcount" matrix using scripts in R[23] provided by the authors of fineStructure. To complement the haplotype based methods, we utilised the population structure detecting ADMIXTURE analysis, as well as calculating F_{ST} between each merged fineStructure cluster. We performed ADMIXTURE analysis using default parameters on the 2,554 combined dataset after pruning the set of common markers for linkage disequilibrium in plink using --indep-pairwise 1000 50 0.2, leaving a set of 88,660 independent common markers. We analysed assuming 2-5 ancestral *k* populations, replicating each *k* value 20 times and choosing the replicate with the highest log-likelihood and lowest cross-validation score. We calculated the F_{ST} between merged clusters using Weir and Cockerham's method[24], using the same individuals and marker set included in the ADMIXTURE analysis.

Additionally we investigated gene flow patterns across Britain and Ireland with Estimate Effective Migration Surface (EEMS) analysis (SI Appendix Supplementary Data 4), and estimated the levels of Runs of Homozygosity (ROH) in each of the merged clusters (see SI Appendix Supplementary Data 3).

Norwegian Ancestry in Britain and Ireland

We performed ADMIXTURE[25] analysis on a combined sample of our British and Irish sample with additional Scandinavian samples (see **Methods: Samples**). We performed this supervised analysis using three populations as surrogate source populations; "England" (the cluster *England*), "Wales" (the clusters *N Wales* and *S Wales*), and Norway (the Norwegian clusters specified in SI Appendix Supplementary Data 6. We first pruned the combined dataset of genetic markers within linkage using the plink[14,15] command --indep-pairwise 1000 50 0.2, leaving 77,864 markers. We performed ADMIXTURE using default parameters with 10 replicates, choosing to analyse the analysis with the highest loglikelihood.

Utilising SOURCEFIND[26], a Bayesian method of estimating ancestry proportions from ChromoPainter haplotype data, we modelled the same target fineStructure clusters as included in the ADMIXTURE analysis as a mixture of all other British/Irish clusters (excluding *Eng-Sco-Ire* due to its admixed nature), and 24 Danish/Swedish/Norwegian clusters (detailed in SI Appendix Supplementary Data 6). We phased with SHAPEIT[18] using human genome build 37, and then performed ChromoPainter haplotype painting, painting all individuals against every other individual. We performed SOURCEFIND analysis with the resultant co-ancestry matrix. We excluded self-copying as an option, choosing not to model excess ancestry as within group ancestry. We tried multiple variations of both number of surrogates and expected number of surrogates used to form a target group, and found changing from 10 and 8, respectively, did not impact the overall results dramatically. We ran the SOURCEFIND MCMC algorithm for 200,000 iterations, discarding the first 50,000 as burnin iterations, sampling every 750 iterations. We chose to model each target as 100 equally sized 'slots' of ancestry drawn from our reference surrogate populations.

Ancient Genetic Links

We explored the shared drift between ancient Icelanders and modern British/Irish and Scandinavians with *D*-statistics, implemented in the *qpDstat* program included in the *admixtools* suit of software[27]. We merged the combined British/Irish/Scandinavian genotype data with 21 Yoruban samples from the Human Genome Diversity Project[16] and the 27 ancient Icelander genotypes with *mergeit* (also included in *admixtools*). We performed PCA using *smartpca*[11,12], with default parameters other than; no outlier removal and projecting the ancient samples onto the genetic diversity of the modern European individuals. We excluded a number of regions with high linkage disequilibrium

whose coordinates on the human genome build 37 are; chr2:135.5Mb-137Mb, chr6:0Kb-750Kb, chr6:25.5Mb-33.55Mb, chr8:7.5Mb-120Mb, and chr11:46Mb-57Mb. This left a total of 209,028 common markers in the final dataset.

Supplementary Data 2 – Final fineStructure Dendrogram

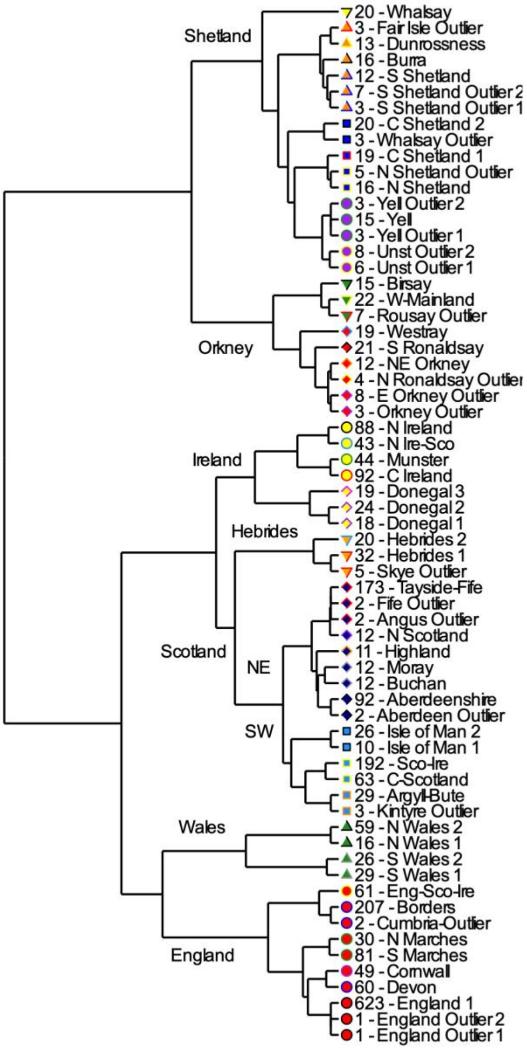

Fig. S1. Final Inferred fineStructure Clustering of 2,554 individuals with regional British or Irish ancestry. The fineStructure dendrogram of the final k = 65 inferred clusters of 2,554 British or Irish individuals. Shown are; the symbols of each k = 42 merged clusters that each final cluster is merged into, the size of each of the individual final clusters, and the name of each of the final clusters.

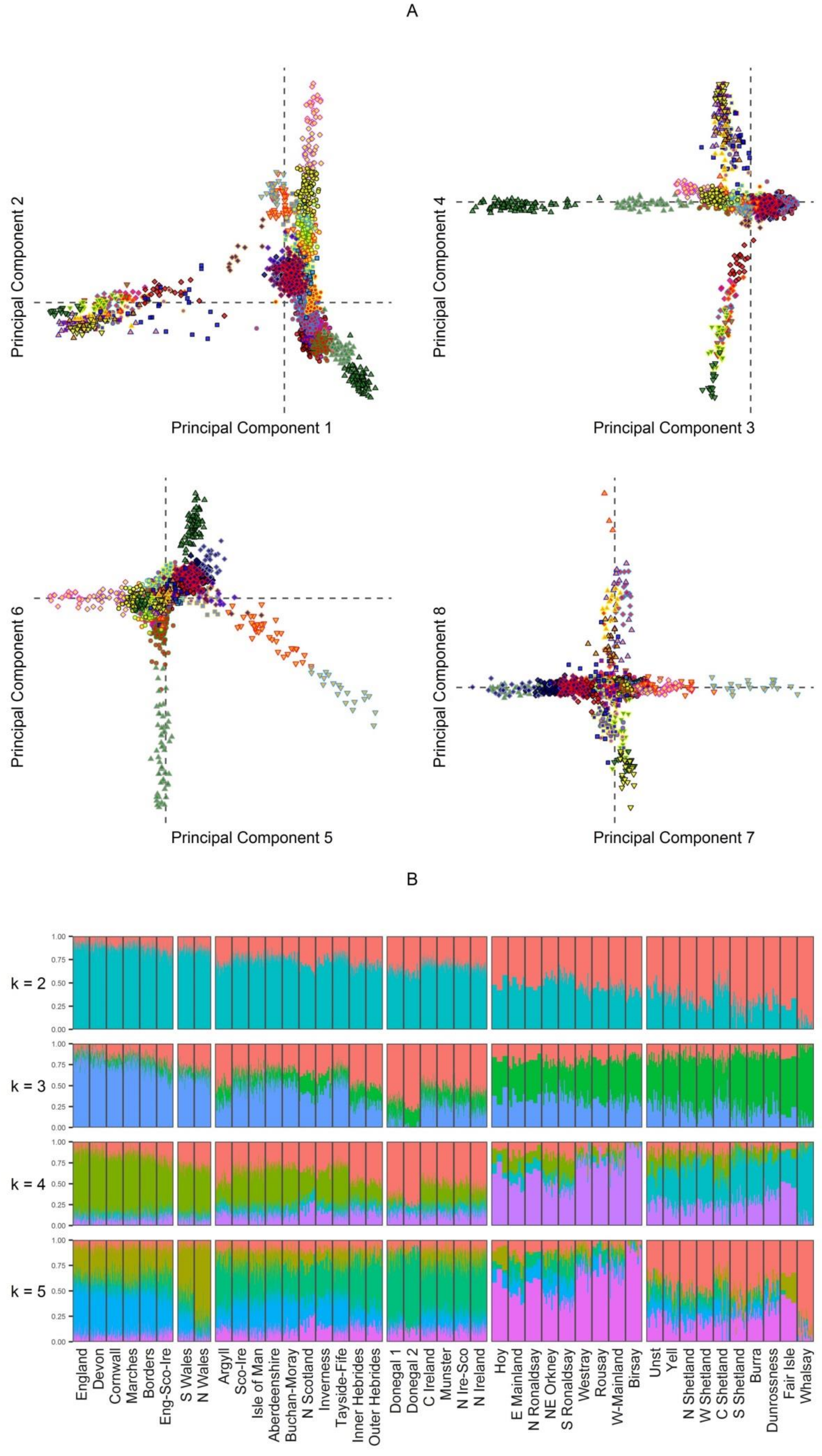
A
Principal Component 1
Principal Component 2
Principal Component 3
Principal Component 4
Principal Component 5
Principal Component 6
Principal Component 7
Principal Component 8
B
k = 2
k = 3
k = 4
k = 5
0.00
0.25
0.50
0.75
1.00
England
Devon
Cornwall
Marches
Borders
Eng-Sco-Ire
S Wales
N Wales
Argyll
Sco-Ire
Isle of Man
Aberdeenshire
Buchan-Moray
N Scotland
Inverness
Tayside-Fife
Inner Hebrides
Outer Hebrides
Donegal 1
Donegal 2
C Ireland
Munster
N Ire-Sco
N Ireland
Hoy
E Mainland
N Ronaldsay
NE Orkney
S Ronaldsay
Westray
Rousay
W-Mainland
Birsay
Unst
Yell
N Shetland
W Shetland
C Shetland
S Shetland
Burra
Dunrossness
Fair Isle
Whalsay

Fig. S2. Genetic Differentiation of 2,554 British and Irish individuals using Principal Components and ADMIXTURE Analyses. (A) The first through to the eighth principal components of the ChromoPainter co-ancestry matrix of British and Irish individuals. Individuals are colour- and shape-coded according to k = 43 merged clusters, using the same scheme as the fineStructure dendrogram (Fig. 1a). (B) The maximum likelihood ancestry profiles of the Irish and British individuals using ADMIXTURE analysis, showing estimates for k populations from 2-5. All panels were plotted in R, using the ggplot2 package.

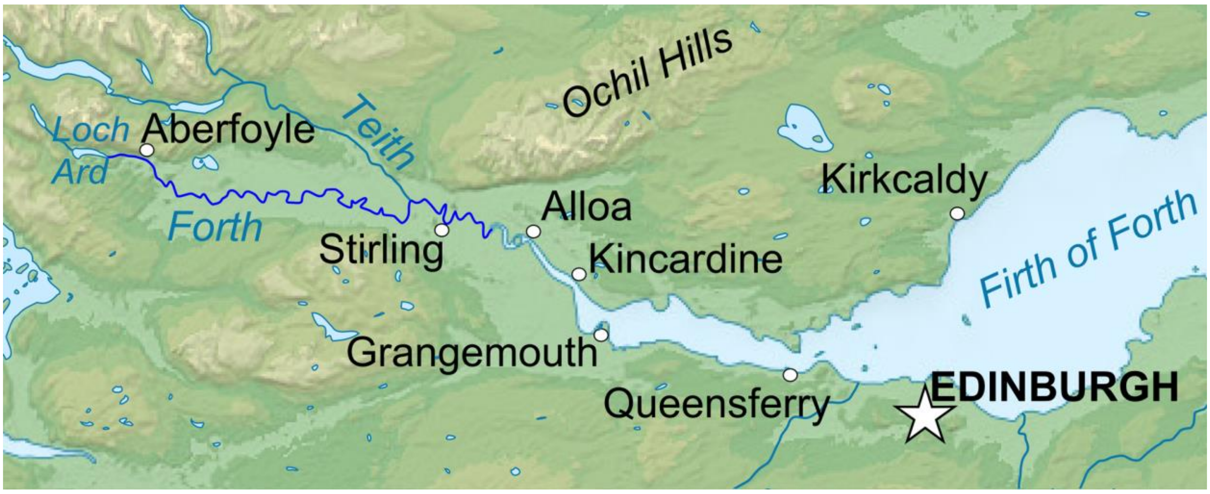

Fig. S3. The geographic course of the River Forth. The course of the River Forth, showing the Firth of Forth. Image is supplied from https://commons.wikimedia.org/wiki/File:River_Forth_course_3.png under the Creative Commons Attribution-Share Alike 3.0 Unported, 2.5 Generic, 2.0 Generic and 1.0 Generic licenses.

Supplementary Data 3 – Runs of Homozygosity

Methods and Results

To supplement the population structure analysis of the British Isles and Ireland, we investigated the levels of homozygosity[28] in each of our merged fineStructure[17] clusters by utilising plink v1.9[14,15] to assess the Runs of Homozygosity (ROH) in each of our clusters. We used the merged dataset used for fineStructure analysis of 2,554 individuals and 341,923 common markers, and with plink we recorded ROH using a window of 1000kb, moving every 50 SNPs, with 1 heterozygous position and 5 missing positions allowed within the window. A ROH was called if it had a minimum of 25 SNPs, a maximum inverse density of 50 kb/SNP, and did not contain a gap of more than 100kb. These parameters we have found in the past to word well in detecting ROH[6,29,30]. We additionally varied the minimum size for an ROH to be called, investigating; 1, 2.5, 5, and 10Mb in minimum length. We recorded the average total ROH for each fineStructure k = 42 merged cluster over each of the minimum lengths (Fig. S4). To calculate the inbreeding coefficient F_{ROH5} we measured the average total ROH > 5Mb a population exhibited and divided it by the length of autosomal genome in our panel of SNPs (2,878,106 kb).

We note that our ancestrally geographically limited sample ascertainment biases us towards an inflated ROH value across clusters as we are sampling individuals with closer recent ancestry than is the case for individuals with ancestry from multiple places. However, this should largely not affect the relative levels between clusters.

Whilst we refer to these results in the main text, we explore them in greater detail here as they represent a comprehensive sample of comparative ROH across the British Isles and Ireland.

We observe the lowest levels of autozygosity in the group of clusters we denote as 'England and Wales'. Most English clusters show the lowest levels of autozygosity in the whole analysis, with the exception of *Cornwall*. *Cornwall* (and Welsh clusters) shows elevated ROH that extend to runs larger than 5Mb – suggesting a degree of genetic isolation which agrees with their respective histories.

Ireland shows slightly elevated levels of ROH compared to the majority of England and Scotland, as has been reported previously[29,31]. We demonstrate that the highest levels of ROH in the settled Irish population (as opposed to the Irish Traveller population, a genetic isolate[29]) are found in the north-western county of Donegal. This level of genetic isolation agrees with our other population structure analyses where Donegal, particularly *Donegal 2*, shows higher levels of differentiation. These analyses taken together suggest Donegal is the most genetically isolated region of Ireland observed to date.

Scotland shows a general level of ROH similar to England, with the exception of a few notable isolates. The north-western cluster *Buchan-Moray* exhibits high levels of both short and long ROH, explaining its isolate nature in our population structure analyses. Clusters originating from the west coast of Scotland (*Argyll*, *Inner* and *Outer Hebrides*) also exhibit high levels of both long and short ROH, indicative of both historical and recent genetic isolation[7,30] and which may explain some of these populations' genetic distinctiveness. Interestingly the Isle of Man, despite being an island in the middle of the Irish Sea, presents no evidence of substantial inflation of autozygosity in our analysis. The Isle Man presents an ROH profile similar to the mainland populations of Scotland, or even England. Lastly, the northern Scottish cluster, *N Scotland* has appeared as a Scottish outlier in our other analyses, showing an elevated proportion of Northern Isles-related ancestry. We demonstrate that whilst there appears to be some evidence of elevated levels of short ROH in *N Scotland*, these levels are not as substantial as found in the west of Scotland. We therefore conclude that the observed structure between this cluster and the rest of Scotland is primarily driven by its Northern Isles ancestry, not simply genetic isolation.

Our findings in both Orkney and Shetland support the Northern Isles as the most genetically isolated location in the British Isles and Ireland, in agreement with previous analyses[7,19]. The average total ROH >1Mb within Orkney and Shetland is high, more than double the average of *England* (52.2Mb and 56.2Mb for Orkney and Shetland, respectively, versus 22.8Mb). All Orkney clusters additionally exhibit high levels of long ROH, indicating both historical and recent small population sizes. We report that the Orcadian region with the highest levels of autozygosity is the island of North

Ronaldsay, which is at the extreme north-east tip of the archipelago and has a small population size. Shetland presents slightly elevated ROH levels compared to Orkney. Fair Isle, found in between Orkney and Shetland exhibits the highest burden of homozygosity, with an average of 24.7Mb of the genome in ROH > 10Mb. Fair Isle has an average F_{ROH5} of 0.034, much higher than the expected F_{ROH5} from offspring of 2nd cousins[7] (0.016), although the sample size is very small. The Shetland cluster with the lowest average ROH across categories (*C Shetland*) predominantly consists of individuals with ancestry from districts near to the largest town of Shetland, Lerwick.

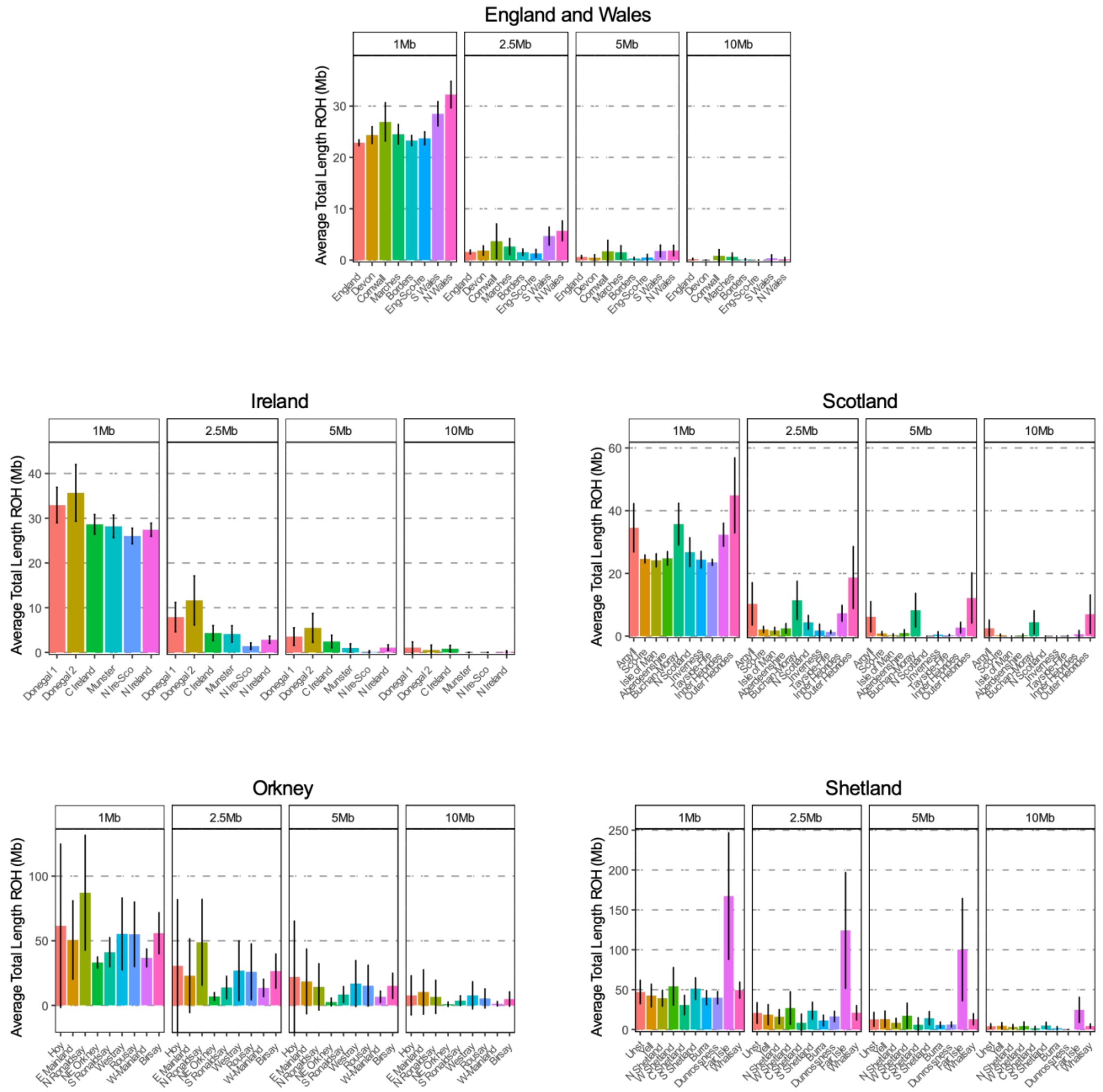

Fig. S4. Extent of Autozygosity Across Britain and Ireland. The mean total length of ROH in 2,544 British and Irish individuals grouped by *k* = 43 merged fineStructure clusters. These clusters are grouped according to geography for ease of reading. The mean total ROH lengths for each cluster included all ROH at least 1, 2.5, 5, or 10Mb in length. Error bars represent the 95% confidence intervals for each cluster. The Y axis is scaled according to geographic region.

Supplementary Data 4 – Estimate Effective Migration Surface Analysis Methods and Results

To supplement investigations into the population structure of Britain and Ireland, we generated an estimate of the effective migration surface of Britain and Ireland using the Estimated Effective Migration Surfaces (EEMS) software[32]. We used a subset of the 2,544 British and Irish individuals, selecting individuals with known birthplaces of great/grandparents (n=2,429). This subset included individuals where an exact longitude and latitude was known, as well as individuals where only the geographic region (such as a county area) was known. For these latter individuals a latitude and longitude was generated at the estimated centre of that region, and all individuals classified with ancestry from that region were described with those coordinates. We utilised the common set of markers used in fineStructure analysis (341,923).

From this subset in plink[14,15] format, we generated a matrix of average pairwise genetic dissimilarities using the program *bed2diffs* included in the EEMS software package. We calculated the latitude and longitude for each individual according to the origin of their recent ancestry. In order to generate the outer boundaries of the analysed area, we utilised an online Google Maps API tool (http://www.birdtheme.org/useful/v3tool.hmtl).

With these data we performed EEMS analysis using the program *runeems_snps*, using 10 initial independent EEMS runs, each with a different random seed. Each of these initial Markov Chain Monte Carlo EEMS runs utilised 2M burnin iterations, and 2M sampling iteration, retaining a sample every 10,000th sampling iteration. Using the replicate with the highest log-likelihood we then started 10 new EEMS runs using that replicate as a starting point, each with a separate random seed. From there each of the ten new runs performed 1M burnin iterations, and 2M sampling iterations, retaining every 10,000th sample iteration. For each EEMS analysis, initial or not, we set the number of 'demes' to 600.

Using all ten final replicates for input, we plotting the results of our EEMS analysis in the statistical software language R[23] (version 3.5.0), using the custom package *rEEMSplots* provided by the authors of EEMS. The average estimated effective migration surface is shown in Fig. 2 of the main manuscript. We show below the posterior probability trace log for all ten final chains (Fig. S5a), the observed vs fitted genetic dissimilarities between pairs of demes (Fig. S5b), and the placement of samples to demes over the estimated effective migration surface (Fig. S5c).

The ten independent EEMS duplicates appear to have all converged, moving around a similar value (Fig. S5a) We find a good fit with the observed dissimilarities between pairs of demes versus the fitted model (R^2 = 0.772) indicating a robust model of migration across the British Isles and Ireland (Fig. S5b). The placement of samples in our analysis is even for most areas in our analysis (Fig. S5c).

The majority of our Scottish individuals are placed at the county area resolution, hence only a number of demes in Scotland are inhabited in our analysis – although many parts of Scotland are actually uninhabited mountainous country. Despite this, we observe patterns of gene flow and gene barriers within Scotland – such as the gene flow barrier that reaches across the Highlands of Scotland, which mirrors the genetic isolation observed there in other analyses. We observe evidence of gene flow between region in the Highlands and the west coast of Scotland. The Highland deme consists partly of individuals whose geographic label was "Highland", with no further information available. This council area includes a very large geographic expanse, and so it is possible that this gene flow reflects an artefact in the EEMS analysis joining the west coast of Scotland to individuals whose ancestry actually originate from the west coast of Scotland but were placed at the latitude and longitude coordinates we defined for the "Highlands" council area.

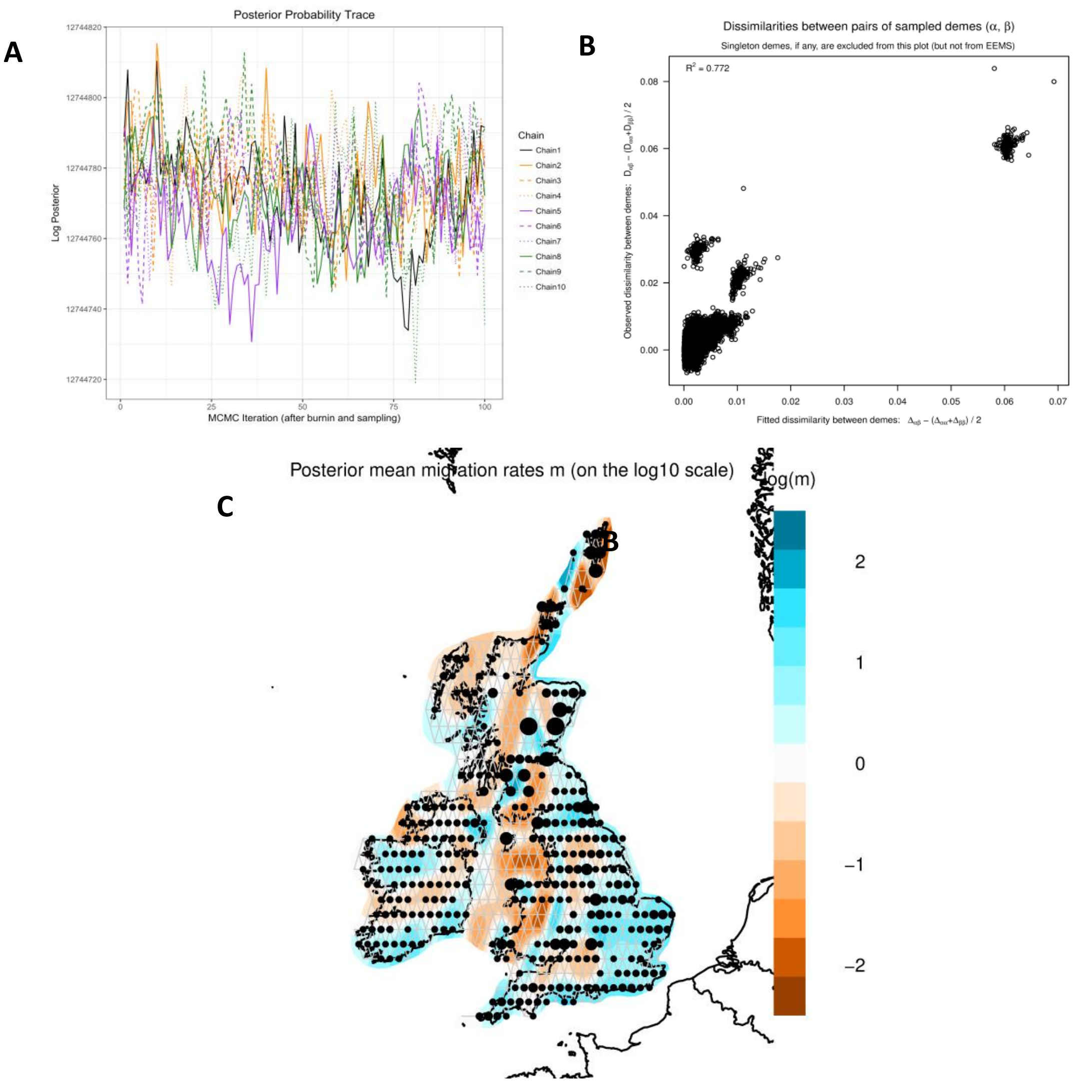

Fig. S5. The diagnostic results of the Estimate Effective Migration Surface (EEMS) analysis. (A) The trace plot of the log posterior probability for each sampled EEMS iteration in each of the 10 duplicate runs or "chains". (B) The scatter plot of the observed versus the fitted dissimilarities between demes, also shown with the R^2 between observed and fitted. (C) The placement of samples (black dots) onto the demes (the vertices of the grey habitat grid), with the migration surface plotted underneath in red/blue. Plots were generated using R. The administrative boundaries were sourced from the rworldxtra R package.

Supplementary Data 5 – Isle of Man Population Structure

Methods and Results

To our knowledge, we present the first high-density, genome-wide analysis of the genetics of the Isle of Man. Previous studies have been limited to either comparison of Manx blood group frequencies[33] or Y-chromosome haplotype frequencies[34] to the rest of the British Isles. This work showed that the Isle of Man presents genetic evidence of both Norse and Germanic influence when considering Y-chromosome diversity[34], thought to reflect its Viking heritage and history of English migration to the island. Today, the Isle of Man continues to have a sizable immigrant population, with about half of the total either native to the Isle or an immigrant from one of the surrounding nations, mainly England[5,35]. The native population itself is considered a 'Celtic' population, formerly speaking the Celtic language Manx, which is related to Irish and Scottish Gaelic, but separate[36].

We update this knowledge with 40 new high-density, whole-genome genotyped individuals, each with 4 grandparents born on the Isle of Man from an existing longitudinal study[5]. Using these samples with 2,514 other British or Irish individuals we performed haplotype based clustering analysis with fineStructure[17]. We identified two clusters out of a final clustering number of k = 65 of sole Isle of Man membership. In the analyse in the main manuscript we chose to merge these two clusters together as we did not have further grandparental birth place information of any of the Isle of Man samples. In this more detailed review of the Isle of Man results we chose to present the two clusters *Isle of Man 1* (n=10) and *Isle of Man 2* (n=26) as separate groups. We demonstrated that they are distinct groups, despite having no access to any further non-genetic information with which to differentiate them. We investigated the hypotheses that this structure is due to Isle of Man-specific drift, or due to different admixture histories in these two lineages.

The two Manx clusters separate out in the dimension reducing t-distributed stochastic neighbour embedding (t-SNE) analysis (Fig. S6a). The smallest Manx cluster *Isle of Man 1* is positioned closest to the mainland British samples in this analysis, plotting with an individual with English ancestry (*Marches*). The *Isle of Man 1*'s closer relationship to England is repeated in principal component analysis (Fig. S6b), where *Isle of Man 1* plots closer to the English pole of principal component 2 and *Isle of Man 2* plots closer to the Scottish/Irish pole. This differentiation could be a result of isolation-by-distance. To investigate the extent of genetic isolation we compared the levels of autozygosity by using the results of our Runs of Homozygosity (ROH) analysis reported in the main results, but comparing the levels between Manx individuals grouped by either *Isle of Man 1* or *2* (Fig. S6c). Whilst *Isle of Man 2* shows a slightly elevated levels of short ROH, we do not find a significant difference of ROH levels between the two groups (t-test between ROH >1Mb, p = 0.747.

To conclude, we find evidence of further structure within the Isle of Man. Unfortunately, without geographic data on the regional origin of the ancestry of our Isle of Man sample we are more limited in our interpretation of these results than the other regions in the British Isles and Ireland that we report. We detect three genetic groups of Isle of Man samples in our analyses. The first are individuals who, despite their extended ancestry from the island, are genetically grouped in English fineStructure clusters (*England*: n=2, *Eng-Sco-Ire*: n=1, and *Marches*: n=1). The second and third Manx groups are individuals grouped solely with other Isle of Man samples. One, *Isle of Man 1*, appears to be genetically closer to mainland Britain, and *Isle of Man 2* shares a greater affinity to Ireland. The structure within the Isle of Man, therefore, appears to be associated with different Gaelic-like and/or differing English ancestry. Further work is needed to elucidate whether this structure is geographically distributed within the Isle, or associated with certain social groups.

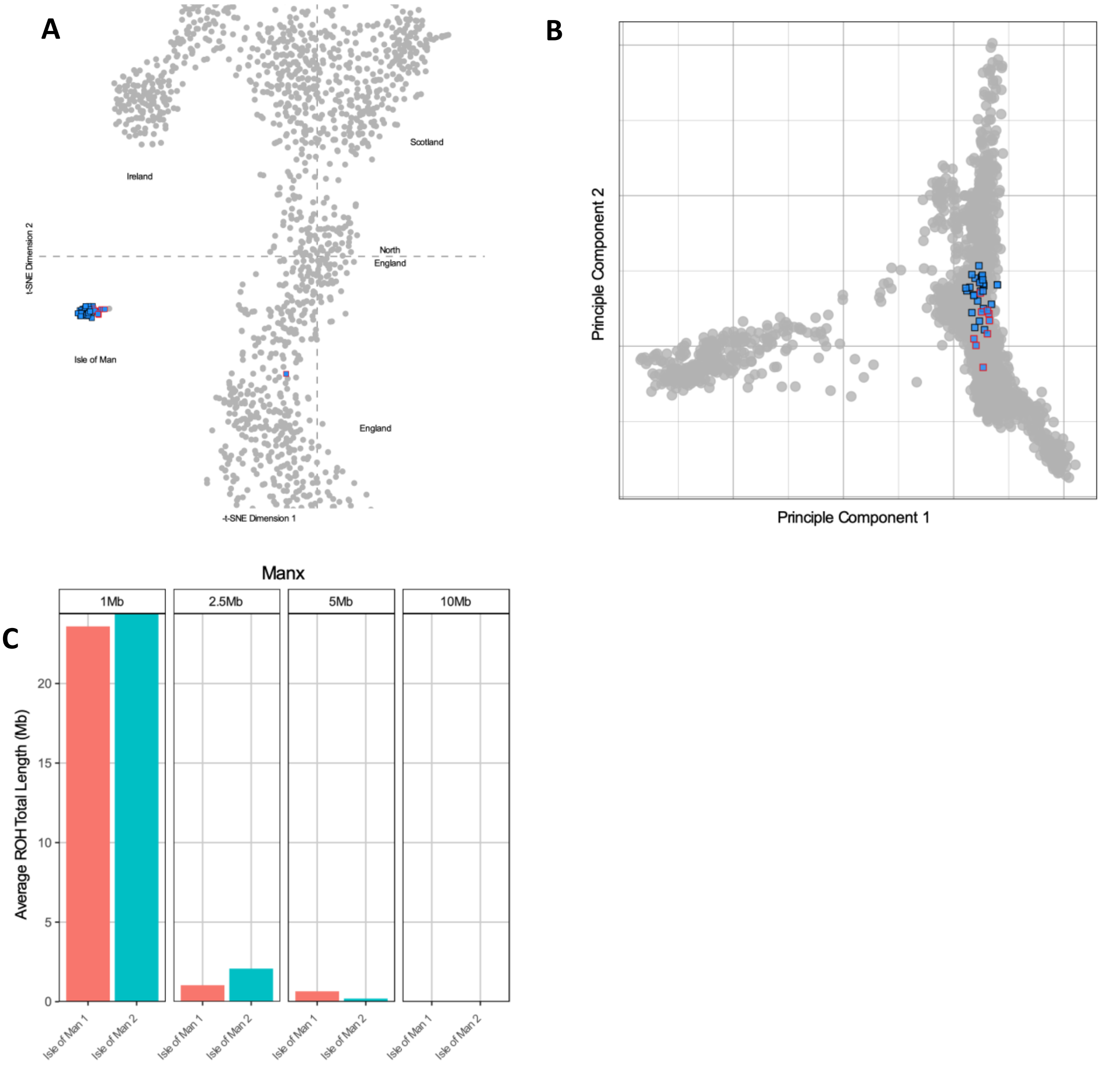

Figure S6. Genetic Differences between Isle of Man fineStructure Clusters. (A) The positions of the Isle of Man clusters *Isle of Man 1* (blue squares red outline) and *Isle of Man 2* (blue squares black outline) in the t-distributed stochastic neighbour embedding compared to British/Irish individuals (grey). (B) The positions of the two Isle of Man clusters relative to other British and Irish individuals (legend the same as panel A) in principal component analysis using principal component 1 and 2. (C) The mean total length of Runs of Homozygosity between the two Isle of Man clusters across four minimum ROH length (1, 2.5, 5, and 10Mb) categories.

Supplementary Data 6 – European Reference Clusters

We utilised previously described European data[10], and grouped according to fineStructure clustering previously performed by our group[6]. Instead of the 52 pan-European clusters, we only considered individuals from the Scandinavian clusters historically associated with Viking activity – i.e. Sweden, Denmark, and Norway. For clarity we record those 24 groups below, noting that the SWE2 and SWE3 appeared admixed in our original analyses and were thus excluded. We show; a brief description of the broad geographic region most individuals within the cluster came from (DESC), the total number of individuals in each cluster (COUNT), and the country of origin of individuals within that cluster (COUNTRY).

NAME	DESC	COUNT	COUNTRY								
			Belgium	Denmark	Finland	France	Germany	Italy	Norway	Poland	Spain	Sweden
SWE1	Stockholm	223	0	0	1	0	0	0	1	0	0	221
SWE4	Sweden	28	0	0	0	0	0	0	1	0	0	27
SWE5	Sweden	33	0	0	0	0	0	0	1	0	0	32
SWE6	Sweden	64	0	0	0	0	0	0	1	0	0	63
SWE7	Sweden	43	0	0	0	0	0	0	0	0	0	43
SWE8	Sweden	28	0	0	0	0	0	0	1	0	0	27
SWE9	Sweden	44	0	1	10	0	0	0	2	0	0	31
SWE10	S Sweden	205	0	2	0	0	0	0	0	0	0	203
SWE11	W Sweden	110	0	0	0	0	0	0	1	0	0	109
SWE12	Sweden	238	0	0	0	0	0	0	0	0	0	238
DEN1	Denmark	316	0	292	0	0	1	0	10	0	0	13
NOR1	N Norway	92	0	0	0	0	0	0	90	0	0	2
NOR2	N Norway	25	0	0	0	0	0	0	25	0	0	0
NOR3	N Norway	92	0	0	0	0	0	0	88	0	0	4
NOR4	NW Norway	47	0	0	0	0	0	0	47	0	0	0
NOR5	S Norway	105	0	0	0	0	0	0	104	0	0	1
NOR6	SE Norway	116	0	3	0	0	0	0	106	0	0	7
NOR7	W Norway	25	0	0	0	0	0	0	24	0	0	1
NOR8	W Norway	50	0	0	0	0	0	0	50	0	0	0
NOR9	W Norway	76	0	0	0	0	0	0	75	0	0	1
NOR10	W Norway	74	0	0	0	0	0	0	71	0	0	3
NOR11	S Norway	70	0	0	0	0	0	0	70	0	0	0
NOR12	S Norway	88	0	0	0	0	0	0	88	0	0	0
NOR13	SW Norway	33	0	0	0	0	0	0	32	0	0	1

Supplementary Data 7 – Ancient Genetic Links

In the main text we explore the links between modern British and Irish genetic regions to ancient Icelanders. We first sought to compare the modern genetic structure we capture to that described by Ebenesersdóttir et al, to determine whether our captured structure dramatically changes the previous ancestry estimates[37]. We combined 4,828 modern British, Irish, Danish, Swedish, and Norwegian genotypes already described in the **Norwegian Ancestry in Britain and Ireland** analysis with 21 Yoruban samples from the Human Genome Diversity Project[16], and the 27 ancient Icelanders[37]. We further excluded markers from five regions of high linkage disequilibrium in our dataset; chr2:135.5Mb-137Mb, chr6:0Kb-750Kb, chr6:25.5Mb-33.55Mb, chr8:7.5Mb-120Mb, and chr11:46Mb-57Mb – leaving a total of 209,028 common markers.

We performed PCA with smartpca[11,12] using the 4,828 modern British, Irish, Danish, Swedish, and Norwegian samples used in the Norwegian Ancestry analysis above – using default parameters without outlier mode and projecting the 27 ancient Icelanders on to the genetic variation of our sample of Britain, Ireland, and Scandinavia (Fig. S7a) using lsqproject. Our sample of Britain, Ireland, and Scandinavia behaves similarly to the sample reported by Ebenesersdóttir et al[37], and is able to differentiate ancient individuals by Norse/Gael ancestry. Continuing with our comparison, using *D*-statistics we tested the affinity of each ancient individual to the 'Gaelic' populations Scotland and Ireland by combining all clusters on those fineStructure branches, and the 'Norse' populations of Sweden and Norway (Fig. S7a). We estimated *D*-statistics using qpDstat from admixtools[27] in the form *D*(Yourban, Ancient Individual; Modern Gaelic, Modern Norse). Our estimates agree with previous estimates[37]. We likewise found good concordance between the second principal component in our principal components analysis and *D*-statistics (Pearson's |r| = 0.99). In the analysis presented in the main results, we grouped our ancient individuals in accordance with overall Gaelic/Norse ancestry into three groups; Gael, Norse, Other. We show the grouping of these individuals by *D*-statistic estimate (Supplemental Figure 4b) and PCA (Fig. S7a).

Whilst in the main text we focus on the genetic affinity of these ancient groups and modern genetic regions in Britain and Ireland, we also investigated the individual affinity of each ancient individual to each modern genetic region (Fig. S8). We observe the same trend shown in Figure 4 of the main manuscript: that ancient Gaelic Icelanders share more genetic drift with British or Irish genetic regions, although some individuals present different affinities. KNS-A1 for example shows a greater affinity to the south of Ireland as opposed to Donegal in the north-west, and the only region for which NTR-A2 shares significantly greater drift with Britain than Scandinavia is in the Isle of Man. Our sample sizes in the more remote regions in north Scotland (represented by *Inverness* and *N Scotland*) prevent us from making accurate inferences on the relationship between ancient Icelanders and this region, and better sampling in the future may help elucidate matters. We do not observe a strong affinity between the ancient Icelanders and Orkney and Shetland, where substantial Norse Viking settlement occured[36]. Whilst this could be a sample size issue, we observe stronger signals in north-western British or Irish clusters which have fewer samples. The substantial Norse admixture detected in Orkney[19] and Shetland may affect *D*-statistic estimates, and indeed using a paired student *t* test we observe significantly smaller estimates of *D* when using Orkney or Shetland as a British proxy than using England (p values $< 1 \times 10^{-6}$), where we expect little or no Norse admixture. Sampling of contemporaneous ancient remains from Orkney and Shetland (and indeed the rest of northern Britain) may help elucidate this in the future.

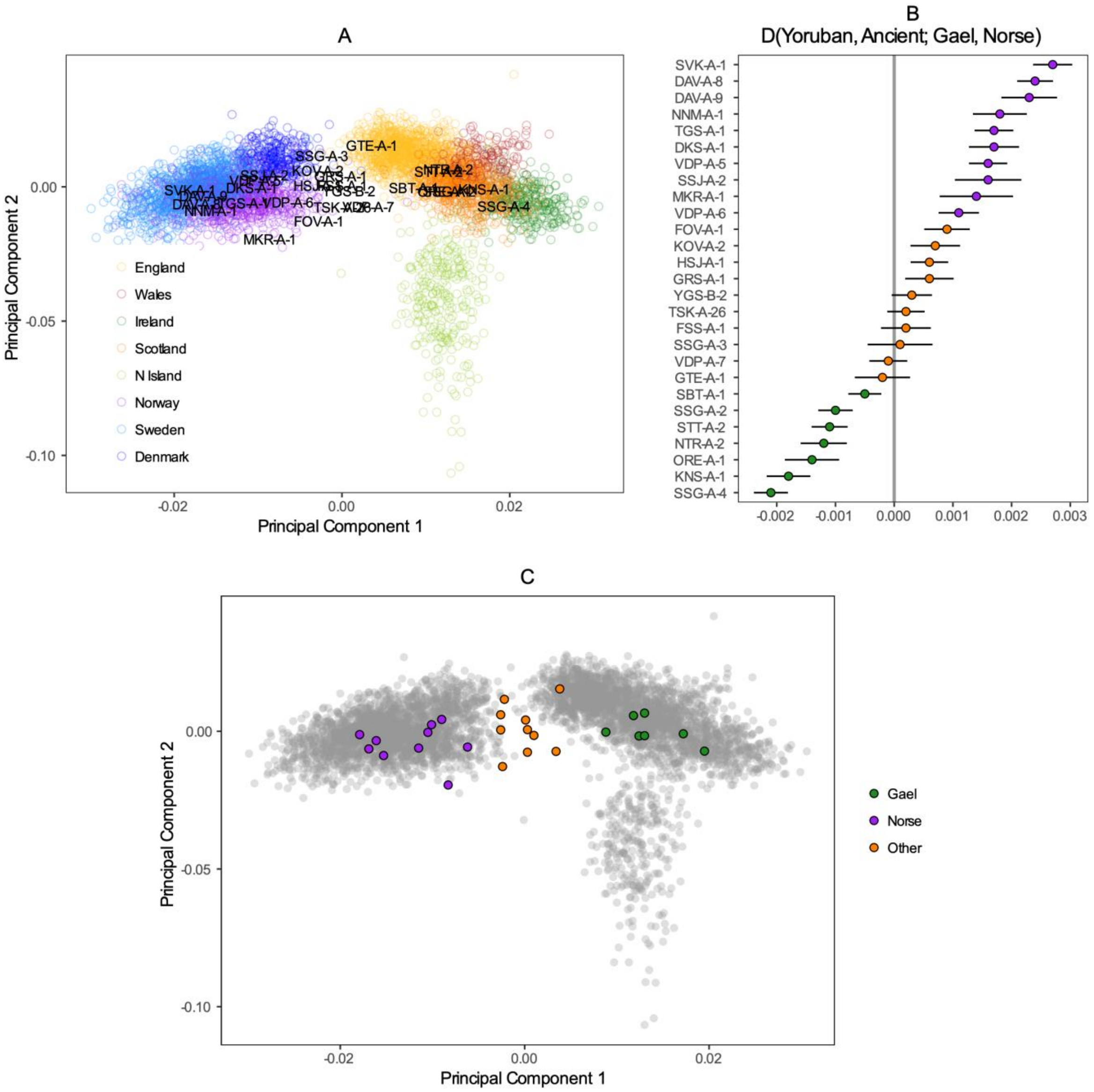

Fig. S7. Broad Ancestry Estimates of Ancient Icelanders. (A) First and second principal components of 4,828 Europeans calculated using *smartpca*[11,12] with 209,028 common markers. Twenty-seven ancient Icelanders are projected onto this variation, whose sample IDs are shown. (B) The *D*-statistic estimates of each ancient Icelander testing *D*(Yoruban, Icelander; Gael, Norse) where 'Gael' is all modern Scottish and Irish samples and 'Norse' is all modern Swedish and Norwegian samples. Points are colour coded according to general group used for subsequent analyses; green is 'Gael', orange is 'Other', and purple is 'Norse'. (C) The first and second principal components as in panel A with colour scheme of ancient individuals same as panel B. All plots were created using the statistical computing language R[23] and the packages ggplots2.

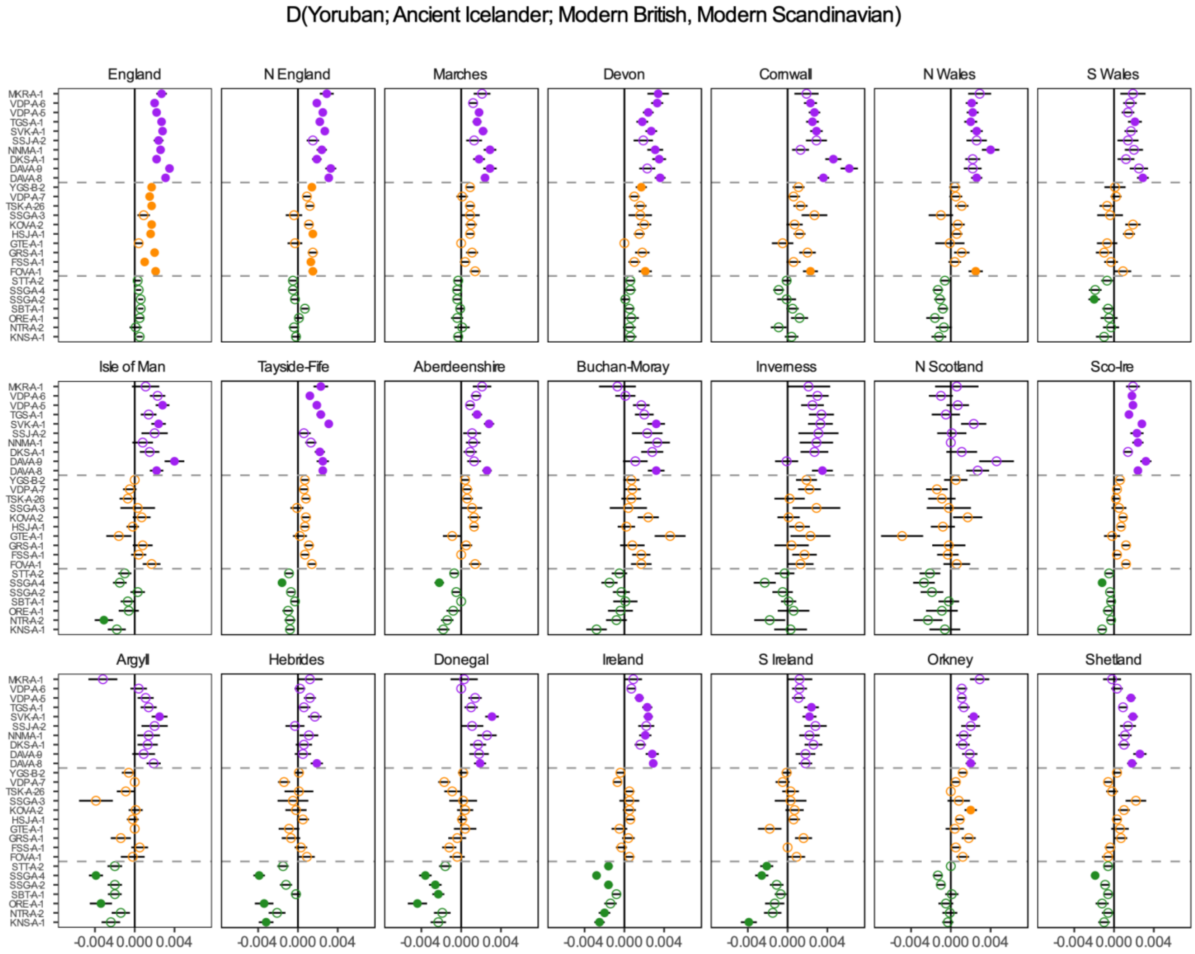

Fig. S8 Shared drift between ancient Icelandic individuals and British or Irish genetic regions. Shown are the individual *D*-statistics using the form *D*(Yoruban, Ancient; British or Irish region, Scandinavia) with standard errors shown. Estimates with a |Z|>3 are shown with filled circles, and those not are shown with a hollow circle. Ancient individuals are grouped according to broad ancestry; Gaelic (green), Norse (purple), or Other (orange). Plots were created using the statistical computing language R[23] and the packages ggplots2.

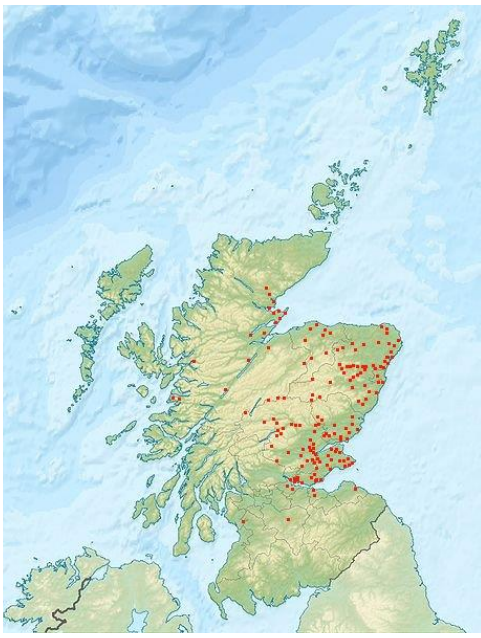

Fig. S9 Map showing the distribution of Pict place names in Scotland. Thought to represent the extent of major Pictish settlement, this map shows the distribution of place names with the Pit or Pett element (e.g. Pittenweem, Pitsligo), meaning a share or portion of land and considered to be of Pictish origin. This map was accessed and reused from Wikipedia under the Creative Commons Attribution-Share Alike 3.0 Agreement, accessed with the following link: https://commons.wikimedia.org/wiki/File:Pit-placenames1.jpg.

Supplementary Discussion – Genetic Structure and Ancestry Across Britain and Ireland

In the main text of our results we discuss the population genetics across the British Isles and Ireland with respect to ancestry both within the Isles and from Continental Europe. Here we discuss in greater detail the geographic distributions, the ancestries, and the wider historical context of the structure that we describe across Britain and Ireland.

Our sample of the British Isles included 1078 English and 131 Welsh individuals sampled from the People of the British Isles Study[4,19] (PoBI). Due to computational limitations we reduced the number of English individuals in this study below that reported in previous analyses of these samples, first by Leslie et al[19] and subsequently by Gilbert et al[6]. Despite this reduction in numbers, the main structure observed previously is recaptured; separating Cornwall, Devon, the Welsh Marches, and the north of England from the central/south majority English cluster. An increased and more representative Scottish sample has moved the cluster of individuals with Borders/north English ancestry (*Borders* here) to the English branch whereas before it had been placed on a Scottish branch[19], perhaps indicating admixture between these two branches. As noted in the main text this cluster's geographic range mirrors the geographic range of the Anglian Kingdom of Northumbria, but also that of the Gododdin. The reduced English sample size in this analysis has also meant that the Cumbria versus Northumbria fineStructure separation (previously suggested[19] to perhaps be indicative of the Brythonic Kingdoms of Rheged and Gododdin respectively) is not observed here. However, the clustering of Cumbria, which was not part of the Kingdom of Northumbria, in this group suggests the earlier Brythonic Kingdoms are driving the signal. Our results in the north of England particularly highlight the importance of both sampling depth and breadth in fineStructure analysis. We note that we do not observe a boundary at the location of Hadrian's Wall, which for centuries marked the limit of the Roman Empire in Britain, and is near the Scottish-English border.

Within our sample of Britain and Ireland, we observe a cluster with English, Scottish, and Irish membership, *Eng-Sco-Ire*, which is related to Borders. The ancestral birth place of these individuals is predominantly found within England, concentrated around the Newcastle and Merseyside regions. These areas saw heavy Irish immigration in the 19th century[38]. We hypothesise, therefore, that this cluster represents individuals of a recent English and Irish admixed ancestry, but this ancestry is distant enough that these individuals otherwise qualified for inclusion in the study. Together with *sco-Ire* and *N Ire-Sco*, there are therefore three clusters with membership from a mixture of British and Irish sources, suggesting at least three different episodes of gene flow between Ireland and Britain.

Whilst we present the first description of the genetic structure and ancestry of the Isle of Man elsewhere (Supplementary Data 5) we discuss the historical context here. The Isle of Man has had a complex history of transitions. The earliest posited language was Manx Brythonic, from the Iron Age, but later Irish influence formed the modern Manx Gaelic language. Subsequently the island experienced Viking rule (indeed many place-names are of Norse derivation, interestingly these vary across the isle[39]), until sporadic English rule in the modern period. The genetic ancestry within *Isle of Man* is about half English, with just over a quarter Irish ancestry, reflecting the Gaelic and English histories. Indeed, four individuals with ancestry from the Isle of Man are instead grouped in the *England* cluster, supporting recent English ancestry within the Isle of Man. The Norse genetic influence on the isle is much smaller than that observed within the other regions formerly under the rule of the Kingdom of the Isles (the Hebrides) or the Jarldom of Orkney (Orkney, Shetland, Caithness, and Sutherland). This suggests that the Norse influence either was diluted by subsequent migrations and conquests, or the genetic impact was minimal as for example with an elite dominance model of rule, whereby numerically inferior newcomers exert control over the people, their customs and language, but have little demographic effect. A larger Manx sample in the future may help to better understand the impact of this complex history.

Previous analyses of population structure within Ireland[6,20] had only sparse coverage in the north-west of the island, County Donegal. A larger sample has identified considerable structure there. Whilst our sampling of the north-west of Ireland is the densest so far, it is an open question to the geographic extent of this cluster along the coast of Donegal and neighbouring Irish counties to the south. As discussed elsewhere, Donegal appears to be the end of a number of genetic clines within

Ireland, including principal component, t-SNE dimensions, ADMIXTURE ancestry proportions, and levels of autozygosity. It appears that this region of Ireland is at the northern end of the Irish genetic continuum. The central-west coast of Ireland (e.g. Connemara) is similarly mountainous like Donegal. Further sampling from this region may, like the case of Donegal, identify other structure that could be related or separate to this novel Donegal structure.

Expanded coverage in Scotland, particularly south-west Scotland, has helped improve interpretation of clusters of shared Irish and Scottish membership. Despite a long and complex history of bidirectional migration and the shared Gaelic language, our haplotype and other analyses suggests one cluster, *Sco-Ire*, represents a movement from Scotland to Ireland, as it branches among the Scottish clusters, specifically with *Argyll*. Among our 162 south-west Scottish samples, 73% belong to this cluster and as this is the most populous part of the country, *Sco-Ire* is therefore likely to be the most common cluster in Scotland. The distribution in Northern Ireland mirrors the distributions of the Plantations of Ireland throughout the 17th century. Thus the cluster will have experienced some genetic isolation by religion from adjacent Irish populations in the intervening centuries. Lowland Scotland appears to be the source of these migrants, in particular Ayrshire, and Lanarkshire. This is supported by the fact that the Ulster dialect of Scots is still spoken in parts of Ulster, mostly in Antrim. Conversely, the *N Ire-Sco* cluster roots among Irish branches. Its distribution in Scotland is therefore likely the legacy of movement from Ulster to Scotland (which *N Ireland* partly follows). We further note that the signal of this Ireland to Scotland movement is not found in the traditional area of the Gaelic Kingdom of Dál Riata in modern Argyll, but rather also around Lanarkshire.

The Ìlich, the people of the Isle of Islay, cluster with those in *Argyll* (including Kintyre), rather than in either Hebridean cluster. Sampling of thus far untested isles such as Barra, Mull, Jura, Coll, Tiree, Colonsay, Bute and Arran will be required to reveal the relationships among them, their degree of isolation and whether they show such fine-scale structure as in the Northern Isles.

The Dark Age Kingdoms of north Britain cast long shadows in the genetics of Scots today. The great northeast to southwest genetic divide which we observe in Scotland reflects remarkably closely the boundaries of Pictland in the northeast and Dál Riata and Strathclyde in the southwest. *Argyll* maps directly onto the Scottish borders of Dál Riata, even including an individual with ancestry from the very north coast of Ireland. Members of *Sco-Ire* map the extent of the Dark Age Brythonic Kingdom of Strathclyde. This P-Celtic Kingdom groups with Q-Celtic clusters (*Argyll*) – reflecting, perhaps, population turnover dating to the conquest of the region by the Gaelic Kingdom of Alba. The distribution of the NE group of clusters (*N Scotland, Inverness, Buchan-Moray, Aberdeenshire, Tayside-Fife*) corresponds tightly with the land of the Picts[36] – evidenced both in the distribution of typical carved Pictish symbol stones and Pictish place-names. The legacy of the later Norse Jarldom of Orkney and its Scandinavian admixture drives the differentiation of both the Northern Isles and the north of Scotland. This Jarldom may further explain the genetic continuum of *N Scotland* (whose region fell under the rule of the Jarldom) between mainland Scotland and the Northern Isles. Ancient DNA will be required to clarify the historical context of these correlations.

The availability of well-documented genealogies from the Northern Isles allowed our sampling of grandparents to be based on parishes, rather than council areas as was the case for the majority of the Scottish samples. Within Orkney and Shetland we observe both incredibly fine-scale structure and the highest proportion of Norwegian-like ancestry in the British Isles or Ireland The structure that we observe within Orkney agrees well with the structure captured by Leslie et al[19] differentiating Westray and the South isles of Orkney from the Orkney Mainland (the largest isle within Orkney). Our sample differentiates the West and East Mainland of Orkney, as well as individual isles such as North Ronaldsay and South Ronaldsay. Interestingly we are able to separate the parish of Birsay from the rest of the West Mainland, despite no obvious barriers to gene flow in that region.

Fair Isle is the most isolated inhabited island in NW Europe, geographically equidistant between Orkney to the south and Shetland to the north. fineStructure analysis groups the island genetically with Shetland, specifically *Dunrossness*. Fair Isle has historically been part of the south Shetland parish of Dunrossness, which at 39 km distance is the closest landmass. Its extreme geographic isolation is reflected in the fact that Fair Islanders have the highest levels of ROH in

Britain or Ireland, more than double the levels of other Northern Isles populations. This is also reflective of the small population size of ‘native’ Fair Islanders, now ~10 individuals.

Within the rest of Shetland we continue to observe both high Norwegian-like ancestry (the highest in all the Isles, peaking in *W Shetland* and *Yell*) and fine scale structure – again differentiating individual islands such as Whalsay, Unst, and Yell. Some of the islands differentiated in this way are < 3 km apart, such as Burra from the Shetland Mainland (or in Orkney, Rousay from the Orkney Mainland) – making this the finest geographic scale genetic structure known to the authors.

Acknowledgments

This work has made use of the resources provided by the Edinburgh Compute and Data Facility (ECDF) (http://www.ecdf.ed.ac.uk/).

Generation Scotland received core support from the Chief Scientist Office of the Scottish Government Health Directorates [CZD/16/6] and the Scottish Funding Council [HR03006]. Genotyping of the GS:SFHS samples was carried out by the Genetics Core Laboratory at the Wellcome Trust Clinical Research Facility, Edinburgh, Scotland and was funded by the Medical Research Council UK and the Wellcome Trust (Wellcome Trust Strategic Award "STratifying Resilience and Depression Longitudinally" (STRADL) Reference 104036/Z/14/Z).

The SCOTVAR study was funded by the UK Medical Research Council (MRC) through a core grant to Prof Alan F. Wright. We are indebted to all the Scottish population participants and general practitioners who joined or facilitated the study. We thank Angie Fawkes and Lee Murphy from the University of Edinburgh Clinical Research Facility for the preparation and genotyping of the DNAs used in the current analysis.

The Viking Health Study – Shetland (VIKING) was supported by the MRC Human Genetics Unit quinquennial programme grant "QTL in Health and Disease". The Orkney Complex Disease Study (ORCADES) was supported by the Chief Scientist Office of the Scottish Government (CZB/4/276, CZB/4/710), a Royal Society URF to J.F.W., the MRC Human Genetics Unit quinquennial programme "QTL in Health and Disease", Arthritis Research UK and the European Union framework program 6 EUROSPAN project (contract no. LSHG-CT-2006-018947). DNA extractions for ORCADES and VIKING were performed at the Edinburgh Clinical Research Facility, where genotyping for VIKING was also performed. We would like to acknowledge the invaluable contributions of the research nurses in Orkney and Shetland, the administrative teams in Edinburgh and the people of Orkney and Shetland.

We are very grateful to all the parents and children who took part in the Isle of Man ELSPAC study, Nic Timpson for helping to facilitate the use of the Isle of Man cohort, the various staff who took part in the various measurements made, and Edna Rolfe in particular for all that she has done to facilitate the study.

We would like to thank participants of the Irish DNA Atlas study for contributing their data, as well as to Dr. Jonna Fay for aiding in the processing and storage of the Irish DNA Atlas DNA samples. The work for part funded by a Career Development Award (13/CDA/2223) from Science Foundation Ireland.

We would like to thank Sir Walter Bodmer for the geographic coordinates of the People of the British Isles (PoBI) samples. This study makes use of data generated by the PoBI project, a full list of the investigators who contributed to the generation of the data is available from the relevant PoBI papers.

This study makes use of data generated by the Wellcome Trust Case-Control Consortium. A full list of the investigators who contributed to the generations of the data is available from www.wtccc.org.uk. Funding for the WTCC project was provided by the Wellcome Trust under award 76113, 085475, and 090355.

We would like to thank Agnar Helgason, S. Sunna Ebenesersdóttir, and Kristjan H. S. Moore for providing the genetic data of the ancient Icelandic Gaels in plink format.

Supplementary Table Legends

Dataset S1
Pairwise F_{ST} Estimates between 42 British and Irish fineStructure Clusters. The pairwise F_{ST} estimates between the merged k = 43 fineStructure clusters, using the Weir and Cockerham method. fineStructure clusters are ordered according to fineStructure dendrogram branch order, and main branches are indicated with dashed lines.

Dataset S2
ADMIXTURE Ancestry Proportions of Northern British and Irish Clusters. The mean average ancestry proportions of a three population supervised ADMIXTURE analysis using England, Wales, and Norway as reference populations.

Dataset S3
Norwegian Ancestry Profiles in Northern British and Irish Clusters. The mean average total genome-wide proportion of haplotypes per tested Northern British or Irish cluster from each reference population.

Dataset S4
Fine-scale Norwegian Ancestry Profiles in Northern British and Irish clusters. The mean average ancestry proportion of haplotypes donated by each individual reference cluster to each tested Northern British or Irish fineStructure cluster.

SI References

1 Smith BH *et al.* Cohort Profile: Generation Scotland: Scottish Family Health Study (GS:SFHS). The study, its participants and their potential for genetic research on health and illness. *Int J Epidemiol* **42**, 689-700 (2013).

2 Smith BH *et al.* Generation Scotland: the Scottish Family Health Study; a new resource for researching genes and heritability. *BMC Med Genet* **7** (2006).

3 Vitart V *et al.* Increased Level of Linkage Disequilibrium in Rural Compared with Urban Communities: A Factor to Consider in Association-Study Design. *Am J Hum Genet* **76**, 763–772 (2005).

4 Winney B *et al.* People of the British Isles: preliminary analysis of genotypes and surnames in a UK-control population. *Eur J Hum Genet* **20**, 203-210 (2012).

5 Goodfellow SA, Rolfe EM & Golding J. Cohort Profile: The Isle of Man Birth Cohort Study. *Int J Epidemiol* **42**, 1246–1252 (2013).

6 Gilbert E *et al.* The Irish DNA Atlas: Revealing Fine-Scale Population Structure and History within Ireland. *Sci Rep.* **7**, 17199 (2017).

7 McQuillan R *et al.* Runs of Homozygosity in European Populations. *Am J Hum Genet* **83**, 359-372 (2008).

8 Bidwell J, Wood N, Clay TM & et al. in *Advances in Electrophoresis.* Vol. 7 (eds Chrambach A, Dunn MJ, & Radola BJ) 311–351 (VCH Press, Weinheim, 1994).

9 Jones RW *et al.* The ALSPAC Study Team. A new human genetic resource: a DNA bank established as part of the Avon longitudinal study of pregnancy and childhood (ALSPAC). *Eur J Hum Genet.* **8**, 653-660 (2000).

10 IMSGC WTCCC2. Genetic risk and a primary role for cell-mediated immune mechanisms in multiple sclerosis. *Nature* **476**, 214-219, doi:http://www.nature.com/nature/journal/v476/n7359/abs/nature10251.html#supplementary-information (2011).

11 Price AL *et al.* Principal components analysis corrects for stratification in genome-wide association studies. *Nat Genet* **38**, 904-909 (2006).

12 Patterson N, Price AL & Reich D. Population structure and eigenanalysis. *PLoS Genet* **2**, e190 (2006).

13 Ebenesersdóttir SS *et al.* Ancient genomes from Iceland reveal the making of a human population. *Science* **360**, 1028-1032 (2018).

14 Purcell S *et al.* PLINK: a tool set for whole-genome association and population-based linkage analyses. *Am J Hum Genet* **81**, 559-575 (2007).

15 Chang CC *et al.* Second-generation PLINK: rising to the challenge of larger and richer datasets. *Gigascience* **4**, 7 (2015).

16 Li JZ *et al.* Worldwide human relationships inferred from genome-wide patterns of variation. *Science* **319**, 1100-1104 (2008).

17 Lawson DJ, Hellenthal G, Myers S & Falush D. Inference of population structure using dense haplotype data. *PLoS Genet* **8**, e1002453 (2012).

18 Delaneau O, Marchini J & Zagury JF. A linear complexity phasing method for thousands of genomes. *Nature Methods* **9**, 179-181 (2011).

19 Leslie S *et al.* The fine-scale population structure of the British population. *Nature* **519**, 309-314 (2015).

20 Byrne RP *et al.* Insular Celtic population structure and genomic footprints of migration. *PLoS Genet* **14**, e1007152 (2018).

21 LJP van der Maaten & GE Hinton. Visualizing High-Dimensional Data Using t-SNE. *J Mach Learn Res* **9**, 2579-2605 (2008).
22 LJP van der Maaten. Learning a Parametric Embedding by Preserving Local Structure. *In Proceedings of the Twelfth International Conference on Artificial Intelligence and Statistics* **5**, 384-391 (2009).
23 R: A Language and Environment for Statistical Computing. (R Foundation for Statistical Computing, Vienna, Austria, 2017).
24 Weir B & Cockerham C. Estimating F-statistics for the analysis of population structure. . *Evolution* **38**, 1358–1370 (1984).
25 Alexander DH, Novembre J & Lange K. Fast model-based estimation of ancestry in unrelated individuals. *Genome Research* **19**, 1655-1664 (2009).
26 Chacón-Duque JC *et al.* Latin Americans show wide-spread Converso ancestry and imprint of local Native ancestry on physical appearance. *Nat Commun* **9**, 5388 (2018).
27 Patterson N *et al.* Ancient admixture in human history. *Genetics* **192**, 1065-1093 (2012).
28 Ceballos FC, Joshi PK, Clark DW, Ramsay M & Wilson JF. Runs of homozygosity: windows into population history and trait architecture. *Nat Rev Genet.* **19**, 220-234 (2018).
29 Gilbert E, Carmi S, Ennis S, Wilson JF & Cavalleri GL. Genomic insights into the population structure and history of the Irish Travellers. *Sci Rep* **7** (2017).
30 Kirin M *et al.* Genomic runs of homozygosity record population history and consanguinity. *PLoS One* **5**, e13996 (2010).
31 O'Dushlaine CT *et al.* Population structure and genome-wide patterns of variation in Ireland and Britain. *Eur J Hum Genet* **18**, 1248-1254 (2010).
32 Petkova D, Novembre J & Stephens M. Visualizing spatial population structure with estimated effective migration surfaces. *Nat Genet* **48**, 94-100 (2015).
33 Falsetti AB & Sokal RR. Genetic structure of human populations in the British Isles. *Ann Hum Biol* **20**, 215-229 (1993).
34 Capelli C *et al.* A Y chromosome census of the British Isles. *Curr Biol* **13**, 979-984 (2003).
35 Simpson Jr S *et al.* The epidemiology of multiple sclerosis inthe Isle of Man: 2006–2011. *Acta Neurol Scand.* **132**, 381–388 (2015).
36 Cunliffe B. *Britain Begins*. (Oxford University Press, 2013).
37 Ebenesersdóttir SS *et al.* Ancient genomes from Iceland reveal the making of a human population. *Science* **260**, 1028-1032 (2018).
38 Neal F. The foundations of the Irish settlement in Newcastle upon Tyne: The evidence in the 1851 census. *Immigrants & Minorities.* **18**, 71-93 (1999).
39 Kneen JJ. *Yn Cheshanght Ghailckagh*. (The Manx Society, 1925).
